# Niche partitioning shaped herbivore macroevolution through the early Mesozoic

**DOI:** 10.1038/s41467-021-23169-x

**Published:** 2021-05-14

**Authors:** Suresh A. Singh, Armin Elsler, Thomas L. Stubbs, Russell Bond, Emily J. Rayfield, Michael J. Benton

**Affiliations:** School of Earth Sciences, University of Bristol, Bristol, UK

**Keywords:** Palaeoecology, Palaeontology

## Abstract

The Triassic (252–201 Ma) marks a major punctuation in Earth history, when ecosystems rebuilt themselves following the devastating Permian-Triassic mass extinction. Herbivory evolved independently several times as ecosystems comprising diverse assemblages of therapsids, parareptiles and archosauromorphs rose and fell, leading to a world dominated by dinosaurs. It was assumed that dinosaurs prevailed either through long-term competitive replacement of the incumbent clades or rapidly and opportunistically following one or more extinction events. Here we use functional morphology and ecology to explore herbivore morphospace through the Triassic and Early Jurassic. We identify five main herbivore guilds (ingestion generalists, prehension specialists, durophagous specialists, shearing pulpers, and heavy oral processors), and find that herbivore clades generally avoided competition by almost exclusively occupying different guilds. Major ecosystem remodelling was triggered multiple times by external environmental challenges, and previously dominant herbivores were marginalised by newly emerging forms. Dinosaur dominance was a mix of opportunity following disaster, combined with competitive advantage in their new world.

## Abstract (web summary)

Terrestrial ecosystems underwent major restructuring through the early Mesozoic, culminating in dinosaur-dominated faunas. Here Singh et al. use jaw morphology to classify tetrapod herbivores into distinct feeding groups and show that their success was shaped by environmental changes and competitive constraints.

## Introduction

Terrestrial ecosystems underwent significant remodelling during the Triassic via floral and faunal turnovers that established many of the structural elements found in modern ecosystems. The preceding Permian-Triassic mass extinction (PTME), 252 Ma, is said to have reset the whole evolution of life^1,2^. Palaeozoic tetrapod survivors of the PTME, such as procolophonid parareptiles and dicynodont therapsids, were superseded by new archosauromorph and mammaliaform clades^3,4^. The turnovers established dinosaurs as the predominant terrestrial tetrapods for the remainder of the Mesozoic, and saw the emergence of key modern groups such as lissamphibians (frogs and relatives), turtles, lepidosaurs (lizards and relatives), crocodylomorphs, and mammals, as well as flies and beetles^3,4^, and several families of ferns and conifers^5^.

The evolution of tetrapods through the Triassic, with the eventual success of the dinosaurs, is a classic example of a biotic replacement^6,7^ for which two explanatory models have been proposed. The first, the ‘competitive replacement model’ (CRM) is that archosauromorphs/dinosaurs outcompeted their rivals because of their more efficient locomotion, respiration, thermoregulation, and/or feeding habits^8–10^. The CRM occurred in two steps, with archosauromorphs first outcompeting and replacing therapsids in the carnivore guild, and then in the herbivore guild, in the Middle and Late Triassic, respectively^8^. The second model, the ‘opportunistic replacement model’ (ORM) focuses on the role of extrinsic environmental perturbations in enabling an opportunistic diversification of archosauromorphs/ dinosaurs following the extinction of competitor groups^11^. New evidence for the ORM is the discovery that the Carnian Pluvial Event (CPE), 233–232 Ma, was a turning point for terrestrial ecosystems; this was a time when climates switched rapidly from arid to humid and back to arid conditions, causing significant extinctions among plants and among the herbivores that depended on them, and further enabling explosive diversification of herbivorous dinosaurs^12,13^. There have been similar debates over competitive and opportunistic models as explanations for many large-scale biotic replacements through geological time^7^, and the Triassic example explored here can act as an exemplar for study of these other events.

Recent work on Triassic tetrapods has changed our understanding of the pattern of biotic replacement but has not resolved the tension between CR and OR models. For example, despite their success, early dinosaurs show no apparent superiority, possessing lower morphological disparity than contemporaneous pseudosuchians (or crurotarsans, crocodile-line archosaurs)^14,15^, and no long-term evolutionary drive or extinction resilience^16,17^. Recent discoveries now suggest an earlier origin for dinosaurs at ~250 Ma in the Early Triassic^18^, and the extinction of the last non-mammaliaform therapsids towards the end of the Late Triassic^19^. This newly extended span of coexistence across the entire Triassic challenges old assumptions of archaic therapsid capabilities. All these points indicate the need for deeper study.

Here, we explore diversity dynamics and eco-morphospaces to investigate the timing of functional and ecological changes between the key clades through the Triassic. We limit our study to the herbivores as they are the basis of the tetrapod food chains, and by far the most abundant animals in each ecosystem. As primary consumers, herbivores constitute the interface between flora and fauna, acting as indicators of wider eco-environmental change^20^. Further, they generally had robust skeletons that are extensively preserved, and the phylogenetics and feeding functions of all key clades have been previously studied. We show that there was considerable stability in niche partitioning and feeding functions among early Mesozoic herbivores, as animals evolved to avoid competitive pressure. Times of stability gave way to turmoil and rapid change when climates and plants changed, but ‘normal rules’ returned as conditions stabilised.

## Results and discussion

### Triassic herbivore ecomorphological feeding guilds

**Fig. 1:**
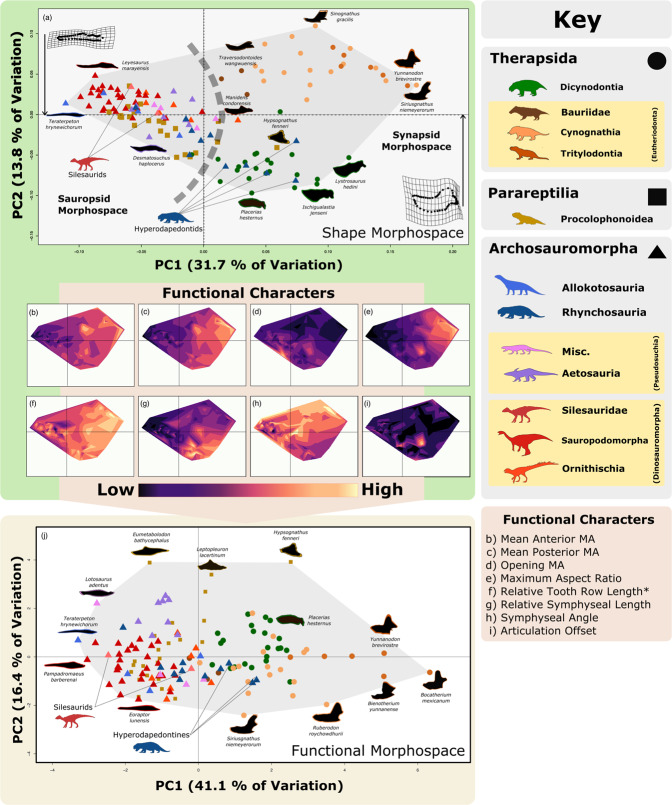
Shape and functional morphospace occupation of early Mesozoic herbivores. **a** Shape morphospace based on geometric morphometric data. **b**–**i** Contour plot of (interpolated) functional character data mapped onto shape morphospace. Increasing magnitude of functional character values indicated by colour gradient from dark to light (scale varies across characters). **j** Functional morphospace based on the above functional characters. Misc., Miscellaneous pseudosuchians. MA, Mechanical advantage. Asterisk indicates tooth row length or length of the mandibular functional surface. *N* = 136 taxa. All silhouettes created by S.S., but some are vectorised from artwork by Felipe Alves Elias (https://www.paleozoobr.com/) and Jeff Martz (United States National Park Service), available for academic use with attribution.

We use herbivorous tetrapod jaws as an ecomorphological proxy and consider variation in both shape and function. Archosauromorphs and therapsids occupy different areas of shape morphospace with almost no overlap (Fig. 1a). The main discrimination between these two clades is along the major axis of variation, principal component (PC) 1, while PC2 discriminates therapsid subgroups, but not the sauropsids, which remain clustered on PC2. This pattern of greater sauropsid conservatism relative to synapsids appears to remain consistent in morphospaces generated from combinations of the first three PCs (Supplementary Fig. 4). Two clades crosscut this general pattern: the areas of morphospace occupied by rhynchosaurs (Archosauromorpha) and procolophonoids (Parareptilia) overlap with other sauropsids as well as with therapsids (Fig. 1a). This functional-ecological discrimination between the two major tetrapod clades, including the ancestors of modern birds and crocodilians on the one hand (archosauromorphs) and mammals on the other (therapsids) helps explain how both clades survived and neither overwhelmed the other, despite evidence for arms races between both through the Triassic^14,16,21^.

Contour mapping of the functional characters (Supplementary Table 1) helps to reveal how jaw shape reflects function (Fig. 1b–i). The sauropsid-therapsid division along PC1 appears closely linked with anterior (Fig. 1b) and posterior (Fig. 1c) mechanical advantage (MA) and maximum aspect ratio (MAR) (Fig. 1e), reflecting biting efficiency and speed, and jaw robusticity. PC2 reflects a more complex pattern and appears to document the opening MA (Fig. 1d), relative symphyseal length (RSL) (Fig. 1g), and articulation offset (AO) (Fig. 1i), reflecting the speed of jaw opening, anterior robusticity, and efficiency of jaw lever mechanics, respectively. These functional characters were used to generate a separate jaw ‘functional’ morphospace (Fig. 1j) in which PC contribution scores indicate that functional PC1 (fPC1) is equally dependent on posterior MA, anterior MA, and MAR, while fPC2 is dominated by the opening MA and AO (Supplementary Table 2). Taxon distribution is more extended along fPC2, but the functional morphospace shows largely the same patterns as seen in the shape morphospace (Fig. 1j and Supplementary Fig. 5). In the functional morphospace, only the rhynchosaurs overlap with therapsids, and they occupy a space between cynognathian cynodonts and dicynodonts, rather than being associated more closely with dicynodonts as in the shape morphospace (Fig. 1a).

Triassic therapsid jaws were highly efficient, granting them relatively high power and speed, as shown by the shape and functional morphospaces (Fig. 1a, j). Therapsids have relatively compressed mandibles (Fig. 1a) that maximise the areas of muscle attachment, increasing MA (Fig. 1b–c). Among therapsids, eutheriodonts developed this characteristic further, diverging from other taxa in terms of the greater compression of their mandibles and the reduced offset between tooth row and jaw joint. This progression continues through the successive positions in morphospace of the bauriid therocephalians, cynognathian cynodonts and tritylodont mammaliamorphs. Relative expansion of the tooth row (Fig. 1f) and development of the jaw musculature supports therapsid optimisation for powerful bites. The more anterior positioning of the adductor musculature in dicynodonts manifests as the highest anterior and posterior MA values of any group with the quadrate-articular jaw joint. Tooth row expansion and low opening MA in eutheriodonts indicates power was directed towards oral processing/mastication, while dicynodont edentulism supports optimisation for a powerful, shearing bite^22^.

Triassic sauropsid jaws were less efficient, but follow similar trends to therapsids in developing comminution ability. Sauropodomorphs and allokotosaurs diverged from these trends, opting for fairly quick but weak bites with relatively large tooth rows to optimise ingestion of vegetation. Aetosaurs, ornithischians and some procolophonoids exhibit morphologies that mechanically improved on the basal morphology of the sauropodomorphs and allokotosaurs, with greater MA and robusticity, although jaw closure was notably slower. This may suggest greater cropping ability and further herbivorous specialisation. Rhynchosaurs show similar trends in developing their jaw musculature, exhibiting MA values (Fig. 1b–d) that converge towards those of therapsids. Leptopleuronine procolophonids are interesting in that their jaws were very stout with slower bite speed and high MA, suggesting they were feeding on very hard/ tough materials. The expansion of the tooth row in aetosaurs, ornithischians and rhynchosaurs suggests they were emulating the eutheriodonts in developing more effective mastication. Consequently, early Mesozoic herbivores can be subdivided broadly by their preference for gut or oral processing^23^. Different groups of therapsids and sauropsids followed common adaptive pathways as specialised herbivores: as phylogenetic contingency combined with ecology to produce convergent forms. This pattern has already been observed among dinosaurs^24^ and our results suggest it runs even deeper in the tetrapod tree.

Regional mapping on the functional morphospace plot (Fig. 1j) shows qualitative groupings that may reflect different functional feeding groups (FFG) or guilds. To quantitatively identify these FFGs, three separate cluster analyses were run using a distance matrix of the standardised functional data. All methods gave similar results with regards to the separation and stability of the cluster groups but disagree over the precise groups (Supplementary Table 5 and Supplementary Data 5). External validation metrics were used to assess how closely the cluster groups corresponded with broad and higher resolution taxonomic groupings (Supplementary Data 14), which highlighted the relatively strong phylogenetic control on mandibular morpho-function (Supplementary Table 6 and Supplementary Data 14). By removing inconsistent taxa and looking for consensus among the three sets of cluster results, we identified five main FFGs: the ingestion generalists (relatively unspecialised), the prehension specialists (stronger, larger bites), the durophagous specialists (slow, powerful bites), the shearing pulpers (that cut and smash plant food), and the heavy oral processors (using teeth to reduce the food). Many sauropsid taxa were recovered within the ingestion generalist FFG, and so the clustering methodology was repeated with the ingestion generalists in an effort to generate higher resolution functional feeding subgroups (FFsG) for use in analysis of potential competition (Supplementary Data 5 and 6). This allowed identification of three additional FFsG within the ingestion generalist group: the basal generalists, tough generalists and light oral processors.

**Fig. 2:**
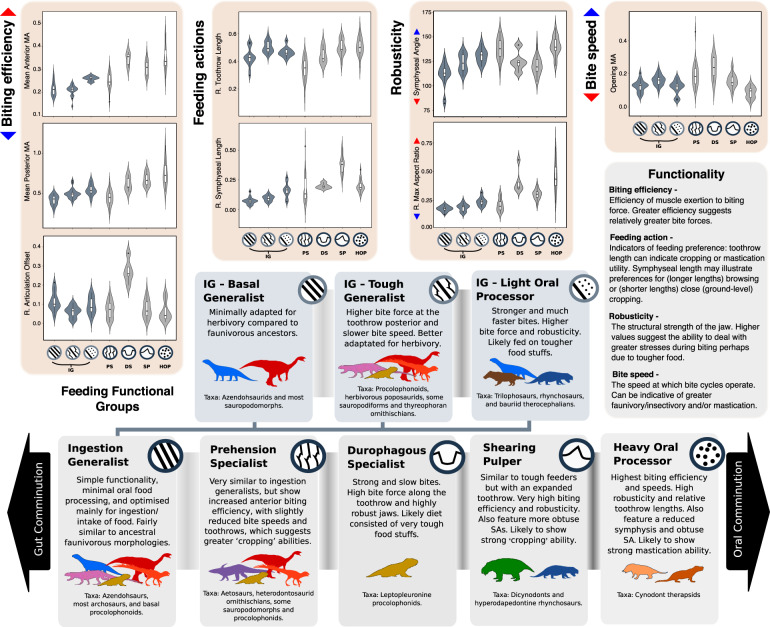
Functional feeding groups. Characteristics of the different functional feeding groups with silhouettes of the taxa that exhibit these feeding modes (see Fig. 1 for silhouette key). Preference of each group for gut or oral processing/comminution of food is indicated. The strength of separation between the groups is illustrated by the darkness of the band connecting each FFG description box. Violin plots show taxon density. Box plots showing median value (centre) and upper and lower quartiles representing the minimum and maximum bounds of the boxes, with whisker illustrating standard deviation. DS durophagous specialist, HOP heavy oral processor, IG ingestion generalist, PS prehension specialist, R Relative, SP shearing pulper, SA symphyseal angle. *N* = 136 taxa. All silhouettes created by S.S., but some are vectorised from artwork by Felipe Alves Elias (https://www.paleozoobr.com/) and Jeff Martz (United States National Park Service), available for academic use with attribution.

**Fig. 3:**
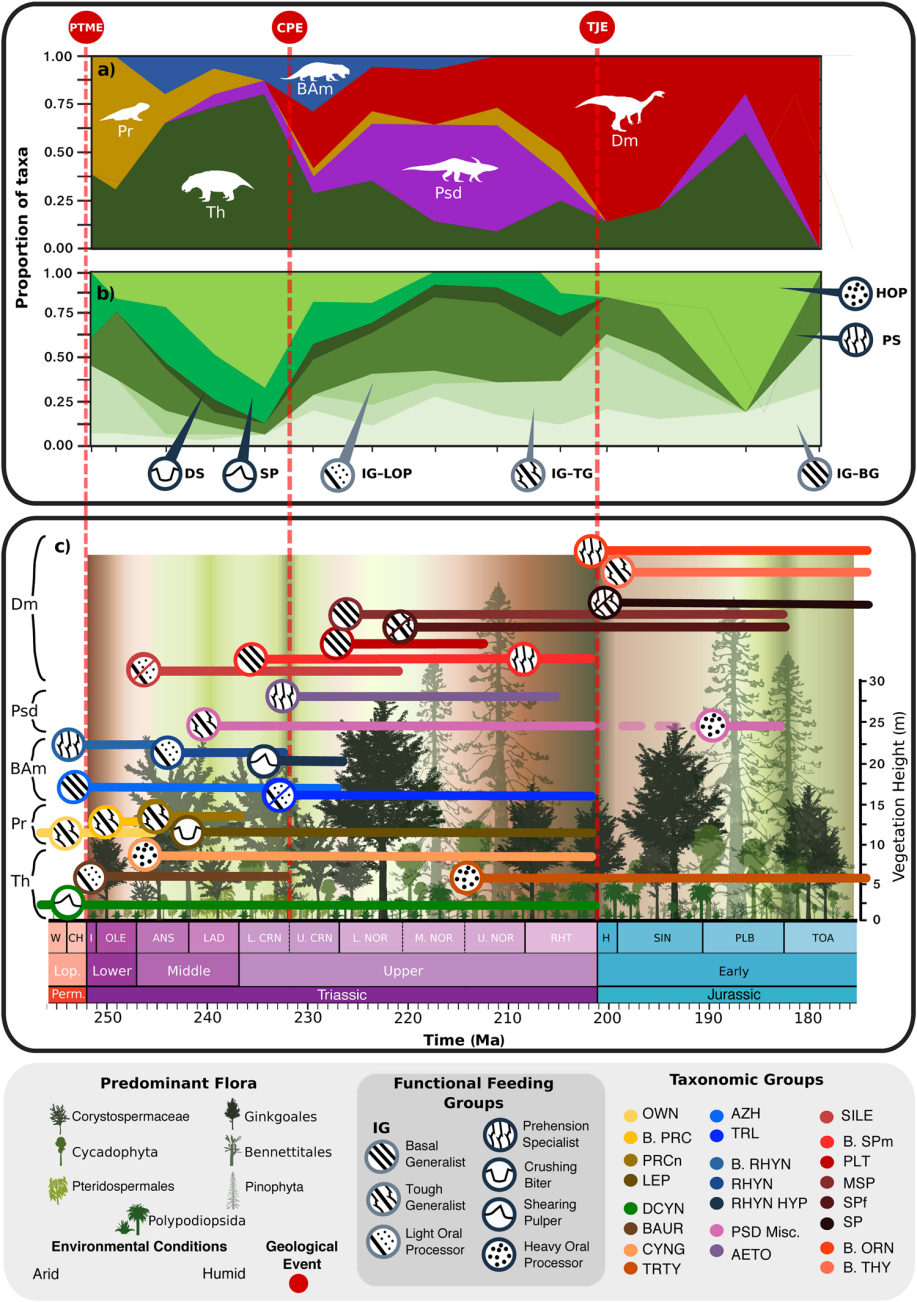
Functional feeding groups of early Mesozoic herbivores through time. **a** The relative species richness of different clades through time. **b** The relative richness of different functional feeding groups through time. **c** Distribution of functional feeding groups across different taxonomic groups and subgroups of herbivores is indicated. Clade and guild changes shown at the midpoints for each stage/substage in panels **a** and **b**. Temporal ranges of the groups are based on first and last fossil occurrence dates, highlighting the span of ecological prominence for each group. Environmental changes from arid to humid shown by background colour gradient. Predominant vegetation^4,60,61^ and characteristic vegetation (relative) height^93,94^ indicated by tree silhouettes. Geological Events: PTME Permian-Triassic mass extinction, CPE Carnian Pluvial Event, TJE Triassic-Jurassic mass extinction, Timebins: ANS Anisan, CH Changhsingian, H Hettangian, I Induan, L CRN Lower Carnian, L NOR Lower Norian, LAD Ladinian, Lop Lopingian, M. NOR Middle Norian, OLE Olenekian, PLB Pliensbachian, RHT Rhaetian, SIN Sinemurian, TOA Toarcian, U. NOR Upper Norian, W Wuchiapingian, Feeding Functional Groups: BG basal generalist, DS durophagous specialist, HOP heavy oral processor, IG ingestion generalist, LOP light oral processor, PS prehension specialist, SP shearing pulper, TG tough generalist, Larger Clades: Dm Dinosauromorpha, Psd Pseudosuchia, BAm Basal Archosauromorpha, Pr Parareptilia, Th Therapsida, Taxonomic Groups: Parareptilia: OWN Owenettidae, B. PRC Basal Procolophonidae, PRCn Procolophoninae, LEP Leptopleuroninae, Therapsida: DCYN Dicynodontia, BAUR Bauriidae, CYNG Cynognathia, TRTY Tritylodontia, Archosauromorpha: ALLOK Allokotosauria, B. RHYN Basal Rhynchosauria, RHYN Rhynchosauridae, RHYN HYP Hyperodapedontinae, PSD Misc Miscellaneous Pseudosuchia, AETO Aetosauria, SILE Silesauridae, B. SPm Basal Sauropodomorpha, PLT Plateosauridae, MSP (non-sauropodiform) Massopoda, SPf (non-sauropod) Sauropodiformes, SP Sauropoda, B. ORN Basal Ornithischia, B. THY Basal Thyreophora, TRL Trilophosauria, All silhouettes created by S.S., but some are vectorised from artwork by Felipe Alves Elias (https://www.paleozoobr.com/) and Jeff Martz (United States National Park Service), available for academic use with attribution.

Dissecting the functional properties within each of the FFGs enables us to determine the likely feeding specialisations (Fig. 2 and Supplementary Data 7) and track their prevalence through geological time (Fig. 3 and Supplementary Data 8 and 9). MA is the main discriminant for our FFGs. The FFGs show that therapsid herbivores fall into three FFGs, and archosauromorphs into two groups. However, the identification of the FFsG shows that archosauromorph morpho-functional differences are more subtle than those present in therapsids, illustrating the varying levels of specialisation and phylogenetic constraints within the two clades. We note that only two FFGs include both therapsids and sauropsids, the ‘shearing pulper’ group, including both hyperodapedontine rhynchosaurs and dicynodonts, and the light oral processor subgroup of the ingestion generalists, which included both archosauromorph rhynchosaurs and trilophosaurs and bauriid therocephalians. Sauropsids show much greater FFG variability within clades than therapsids, where feeding mode is largely common to the entire clade (Fig. 2 and Supplementary Data 5 and 6). This may reflect greater ecological diversification within sauropsid clades as a result of being relatively unspecialised compared to contemporaneous therapsid herbivores, which were already quite specialised at the onset of the Mesozoic. This contrast in specialisation granted sauropsids greater freedom to diversify across different guilds, despite therapsids possessing more mechanically efficient jaws (Fig. 2).

### Niche partitioning and competition avoidance

Were different clades of herbivores apparently competing for the same resources and in the same way? It seems not. We find that differences in jaw morphology are highly constrained by phylogeny and our FFGs do closely reflect phylogenetic groupings. Such phylogenetic structuring does not preclude meaningful functional interpretation of our FFGs to study divergent feeding strategies;^25,26^ this simply reflects that morphology and thus functionality is highly controlled by phylogeny. The distinction between the areas of morphospace occupied by therapsids and archosauromorphs (Fig. 1a) represents their fundamentally different feeding priorities, in which archosauromorphs optimised prehension and therapsids optimised comminution. Therapsids appear to have consistently enhanced biting power, possessing greater MA than most sauropsids, and this may reflect differences in the primary jaw adductor musculature of sauropsids (pterygoideus) and therapsids (adductor mandibularis)^27^. Sauropsid jaw mechanics are less efficient compared to therapsids, but it is clear that sauropsids, particularly the archosaurs achieved significantly larger body sizes than therapsids^16^. Therefore, it appears that sauropsids favoured increasing their bite forces through boosting jaw muscle mass and the absolute power involved, rather than improve efficiency. Their separation in morphospace suggests broad-scale niche partitioning between members of these two clades, guided in part by phylogenetic constraint. Nonetheless, our patterns of shape and functional morphospace occupation show how both groups converged from basal amniote (faunivorous) morphologies^28^ towards a common amniote-specific form of herbivory^29^.

**Fig. 4:**
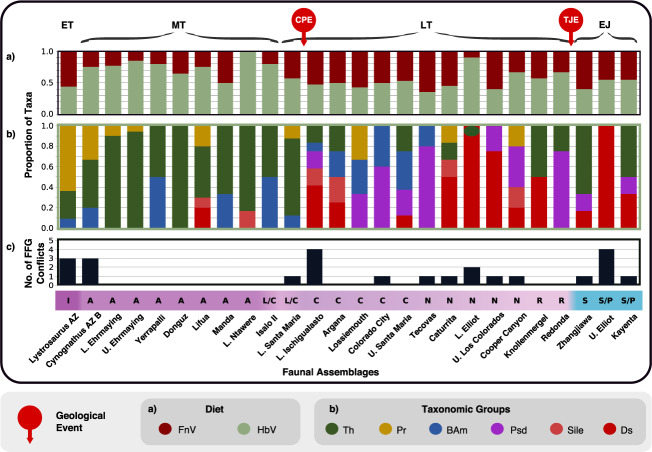
Relative faunal abundances and potential competitive trophic conflicts within early Mesozoic assemblages through time. **a** The relative abundance of faunivores and herbivores. **b** The relative species richness of different therapsids and sauropsid clades. **c** The number of feeding functional group (FFG) conflicts in each assemblage. AZ Assemblage Zone, L Lower, No Number, Geological Events: CPE Carnian Pluvial Event, TJE Triassic-Jurassic mass extinction, Epochs: EJ Early Jurassic, ET Early Triassic, LT Late Triassic, MT Middle Triassic, Timebins: A Anisian, C Carnian, I Induan, L/C Ladinian/Carnian, N Norian, R Rhaetian, S Sinemurian, S/P Sinemurian/Pliensbachian, Diet: FnV Faunivores, HbV Herbivores, Taxonomic groups: BAm Basal Archosauromorpha, Ds Dinosauria, Pr Parareptilia, Psd Pseudosuchia, Sile Silesauridae, Th Therapsida.

At the level of FFGs, minimal overlap between the various therapsid and archosauromorph clades confirms that these herbivores were not in competition for most of the early Mesozoic, contrary to the competitive model (Fig. 3). When our FFGs are applied at ecosystem level for different localities (Fig. 4; Supplementary Data 11 and Supplementary Table 6), we find that most co-occurring taxa belonged to different FFGs. Examples of coexisting herbivores with the same feeding functionality (Supplementary Table 7), and thus possibly competing, include procolophonids, bauriids and rhynchosaurs in the Early Triassic, hyperodapedontine rhynchosaurs and dicynodonts in the Lower Ischigualasto Formation (Carnian), and within dinosaur-dominated assemblages of the latest Triassic and Early Jurassic (Fig. 3), which is expected as most of these dinosaur groups have been shown to employ similar ‘orthal’ jaw mechanics^30^. Widespread morphological dissimilarity suggests that high herbivore diversity in the Santa Maria, Ischigualasto, and Lossiemouth formations (Fig. 4) was sustained by niche partitioning, which enables ecologically similar taxa to coexist by diverging from each other in their demands on resources^31,32^. The subdivision of resources by specialisation towards separate niches minimises resource competition, whilst boosting feeding efficiency, and thus the chances of survival^33–35^.

Our FFGs are broadly defined, so even these examples of possible competition may be exaggerated. The further identification of large subgroups within the ingestion generalist FFG (Fig. 2) highlights this, as use of these subgroups dramatically reduced the occurrences of potential trophic conflict (Supplementary Data 11). Additionally, in the Carnian examples, the kannemeyeriiform dicynodonts were much larger^36^ and lacked the dental plates of rhynchosaurs^37^. These two clades may well have specialised on different plant food while coexisting within the same broad feeding guild. Further, among the Late Triassic herbivorous dinosaurs that also coexisted within broad feeding guilds (Fig. 3), niche partitioning has been noted already among sauropodomorph dinosaurs, expressed in their body size^38^ and postural disparity^39^. Further evidence of tetrapod niche differentiation may be found in their dentition^40^, body size^41^, limb anatomy^42^, and even spatiotemporal behaviour^43^. Therefore, other aspects of ecology may support divergent trophic strategies and the avoidance of competition within these groups, although further comparative studies are needed. Competition between Early Triassic diapsids is more convincing as there are greater levels of coexistence, similarities between sizes, and abundances where found together (Supplementary Data 10).

### Temporal trends: changing of the guilds

**Fig. 5:**
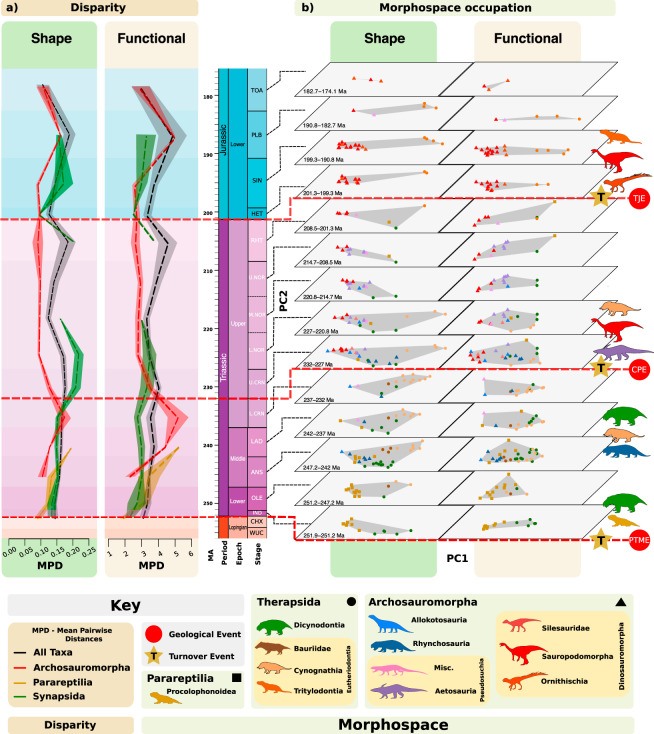
The shape and functional disparity and morphospace occupation of early Mesozoic herbivores through time. **a** Shape (Procrustes variance) and functional (sum of variance) disparity of Archosauromorpha, Therapsida, and Parareptilia, with standard error bands. **b** Shape and functional morphospace time-slices at stage and substage levels. Major extrinsic, environmental events are shown by the dashed red line. Faunal turnovers are highlighted by stars. Misc Miscellaneous pseudosuchians, MPD Mean Pairwise distances, PTME Permo-Triassic mass extinction, CPE Carnian Pluvial Event, TJE Triassic-Jurassic extinction, Timebins: ANS Anisan, CHX Changhsingian, HET Hettangian, IND Induan. L, CRN Lower Carnian, L. NOR Lower Norian, LAD Ladinian, M. NOR Middle Norian, OLE Olenekian, PLB Pliensbachian, RHT Rhaetian, SIN Sinemurian, TOA Toarcian, U. NOR Upper Norian, WUC Wuchiapingian, All silhouettes created by S.S., but some are vectorised from artwork by Felipe Alves Elias (https://www.paleozoobr.com/) and Jeff Martz (United States National Park Service), available for academic use with attribution.

Patterns of shape and functional disparity through geological time (Fig. 5a) generally show near reciprocal traces for therapsids and archosauromorphs—when values for one clade are trending upwards, those for the other are trending downwards. This is particularly apparent in the lower Carnian and Rhaetian. However, this pattern appears to vanish in the Norian, possibly due to poor sampling of the therapsids. Crossovers occur at the times of the Carnian Pluvial Event, 233 Ma, and in the aftermath of the Triassic-Jurassic mass extinction (TJE), 201 Ma. Both metrics broadly agree, showing rising archosauromorph shape and functional disparity through the Early and Middle Triassic, and then higher values for therapsids through most of the Late Triassic, and equivalent values in the Early Jurassic. Interestingly, this concordance breaks down in the Early Jurassic as a disconnect appears within therapsids (tritylodonts), with high shape disparity producing lower functional disparity.

Dividing the shape and functional morphospaces temporally as stacked plots shows more detail of how different herbivorous clades waxed and waned (Fig. 5b). Herbivore guilds in the Early Triassic were dominated by procolophonoids and dicynodonts. During the Middle Triassic, parareptile disparity rose as the Early Triassic disaster fauna was complemented by new groups such as the gomphodont cynognathian cynodonts and archosauromorph allokotosaurs and rhynchosaurs. Archosauromorph disparity also increased as diversity increased with the emergence of new groups with new forms and functions, such as the rhynchosaurs and allokotosaurs. Therapsid disparity remained stable with the diversification of many morphologically similar kannemeyeriform dicynodonts masking the new diversity of cynodonts.

Near the beginning of the Late Triassic, the CPE marked a substantial change, as rhynchosaurs and dicynodonts disappeared or reduced to very low diversity and abundance, and archosauromorph herbivores took over^11–13^. These were initially aetosaurs and sauropodomorph dinosaurs and, while expanding in diversity, their disparity declined (Fig. 5a) because new taxa were morphologically conservative, exhibiting limited variance and emerging within the existing morphospace of each respective clade (Fig. 5b). At the same time, all other herbivore clades declined, with remaining (parareptile and dicynodont) taxa shifting towards the extreme edges of their former morphospace occupancy. Cynognathians also dwindled in the early Norian. This transition within the herbivore guilds marks a shift from oral to gut processing among the majority of large terrestrial herbivores^23^ (Figs. 2, 3, and 5b).

During the Rhaetian, herbivore diversity and disparity declined with only dinosaur and mammalian herbivores surviving into the Jurassic. Both groups underwent morphological and taxonomic radiations in the Early Jurassic, with dinosaurs and mammals typically occupying the roles of large and small herbivores, respectively. There was also a brief reappearance of pseudosuchian herbivores. We note that through the course of the early Mesozoic, sauropsid and therapsid morphospace became increasingly distanced from each other, with further comparison of the distances between therapsid and archosauromorph morphospace centroids showing that this separation accelerated at the onset of the Late Triassic (Supplementary Table 12).

At epoch scale, NPMANOVA identified significant shifts in morphospace occupation between the Early and Middle Triassic (shape and function: *p* = 0.02). At stage level, only the Olenekian-Anisian transition shows a significant shift in both shape and functional morphological diversity (shape: *p* = 0.009, function: *p* = 0.007) (Supplementary Table S14). These results denote the distinct shift from disaster faunas through the Early Triassic, marked by repeated climate perturbations, to the more stable conditions of the mid-Anisian onwards and faunal recovery from the PTME^44,45^. The transitions between the lower Carnian-upper Carnian and Sinemurian-Pliensbachian were identified as being significant to shape but not function (*p* = 0.01 and 0.03) (Supplementary Table 14). These results for the Carnian are tantalising and tentatively highlight the impacts of the CPE as an important macroevolutionary event^13^. Furthermore, at the *p* < 0.1 significance level, the functional differences between these two transitions are recovered as significant (*p* = 0.06 and 0.05), as well as the Pliensbachian-Toarcian transition (*p* = 0.1). However, it must be noted that if a Bonferroni correction is applied, we are unable to recover any significant results for stage transitions.

We recognise a repeated pattern in the replacements in herbivore guilds that coincided with the three crisis events: (1) In the case of the PTME, so many clades had been entirely wiped out by the severity of the extinction that the few species of procolophonoids and dicynodonts that survived^2,46^ would likely have occupied a much reduced ecospace relative to the latest Permian. While procolophonoids began to decline in the Anisian, dicynodonts radiated alongside new rhynchosaurs and cynognathians. These clades came to dominate Middle Triassic herbivore guilds. (2) The CPE hit these dominant groups hard, with survivors hanging on in the peripheries of their former morphological and functional space (Fig. 5b). Through the Norian and Rhaetian, these taxa became further confined to extreme areas of morphospace, whilst new archosaurian herbivores radiated. (3) The TJE was a major blow for the last procolophonoids, dicynodonts and cynognathians, (rhynchosaurs having already succumbed to extinction in the early Norian), as well as the aetosaurs, which had been important elements within Norian faunas (Fig. 3c). We find that these taxa actually began to decline during the Norian (Fig. 5). The decline in these formerly dominant groups is mirrored by expansion of new dinosaur and mammalian herbivore clades. Despite also suffering through the latest Triassic, both groups radiated in the Early Jurassic, moving into space vacated by aetosaurs and cynognathians, respectively. The Early Jurassic fossil record is limited, but total herbivore shape and function space were later refilled by sauropodomorph and ornithischian dinosaurs, as well as new mammalian clades.

**Fig. 6:**
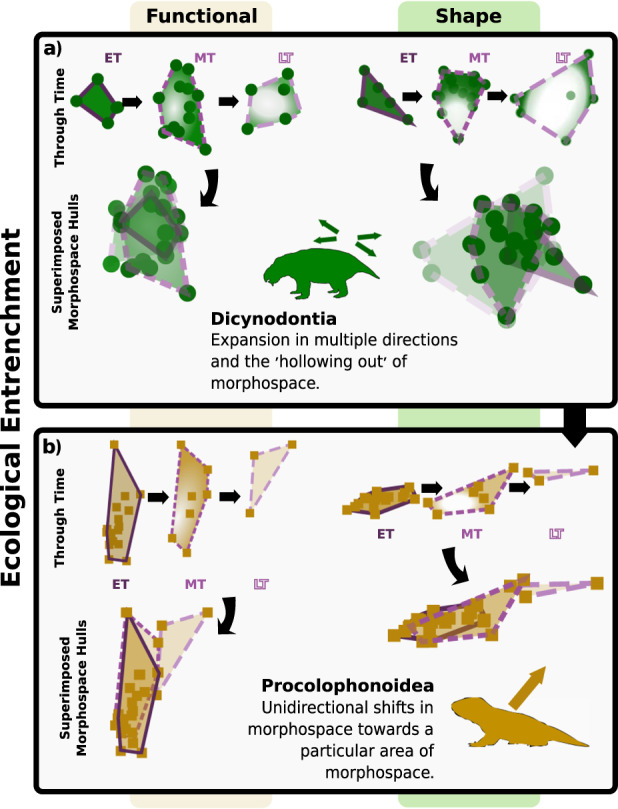
Ecological entrenchment illustrated using morphospace occupation through time. Two main patterns of ecological entrenchment observed within early Mesozoic herbivores: **a** The increasing specialisation of taxa concentrates diversification at the peripheries of morphospace, leading to the hollowing out of total morphospace occupation (as shown here in dicynodonts). **b** A unidirectional shift showing clear specialisation towards a specific eco-morphology (as shown here in procolophonoids). Morphospace areas from each epoch are shown in isolation and overlaid over each other to highlight the shifts through time. Epochs: EJ Early Jurassic, ET Early Triassic, LT Late Triassic, MT Middle Triassic, All silhouettes created by S.S., but some are vectorised from artwork by Felipe Alves Elias (https://www.paleozoobr.com/) and Jeff Martz (United States National Park Service), available for academic use with attribution.

This pattern of marginalisation seen in both shape and function space (Fig. 5b) documents how stressed clades apparently ‘retreat’ into specialised niches at the periphery of their former occupancy. Sampling issues may confound observation of this pattern at stage level, but epoch-level comparisons of morphospace occupation highlights this pattern of declining disparity in certain clades through the Triassic (Fig. 6). This is seen three times through the Triassic and Early Jurassic, as the last parareptiles, rhynchosaurs and dicynodonts were pushed to peripheral positions in shape and function space after the rigours of the three mass extinction events (PTME, CPE, TJE). Likely then, the last survivors of each of these clades had become trophic specialists. As specialists, dicynodonts, hyperodapedontine rhynchosaurs and leptopleuronine procolophonids were potentially more constrained than the new archosaur herbivores in shifting their diets towards the new prevailing flora. Survivors became further entrenched within specialist niches and rare following the crises. In becoming highly specialised, these groups were forced along an evolutionary ratchet^47^ that amplified extinction risk as the environment changed and those niches disappeared. Specialists may outcompete generalists where high quality resources are readily available and stable^48^.

Consequently, this trophic specialism in combination with reduced abundance suggests this ‘ecological entrenchment’ is possibly correlated with geographic retrenchment to where preferred resources remained abundant, with the reduction in numbers and geographic spread exacerbating extinction risk^49,50^. ‘Marginal’ morphospace occupation may be followed by further restriction of MO to a smaller subset of morphospace (Figs. 5b and 6), which may relate to further ‘hyper-specialisation’ or perhaps the ongoing loss of refugia as conditions became increasingly adverse. Nonetheless, poor sampling is an acute issue, particularly as these clades approached extinction in the Late Triassic, so further study is required to test these tentative interpretations. Ecological entrenchment may have served to minimise competitive pressures and prolong survival in the face of increasingly heterogenous environmental conditions and new competitors that were able to better exploit predominant plant resources.

### Extrinsic controls on herbivore macroevolution

Triassic climates oscillated between acute humid and extended dry phases^51^, and these fluctuations triggered widespread and significant remodelling of terrestrial floras^5,52^. Floral turnovers coincided with pulses of change in herbivore guilds. The transition from palaeophytic to mesophytic plant assemblages through the Ladinian and Carnian^52^ coincided with reduced morphospace packing by non-archosaurian herbivores (Figs. 3c and 5b). Herbivore functional diversity among dinosaurs and pseudosuchians expanded following the CPE. Widespread wetter climates in the CPE^53,54^ may have triggered radiations of Bennettitales, Gnetales, and modern ferns and conifers^5^, associated with the expansion of archosaurian herbivore diversity and taxon density within archosaur morphospace, which counter-intuitively reduced archosaur disparity (Fig. 5a). Increased morphospace packing by archosaurian herbivores (Fig. 5b) tentatively suggests that the increased prominence of some gymnosperms as arid conditions returned in the Norian^52^ may be linked to the survival of archosaur herbivores, particularly sauropodomorphs through the Carnian-Norian transition, whilst other herbivore groups perished.

The CPE was critical in triggering the decisive switch from dominance by therapsids as herbivores to the real beginning of the ‘age of dinosaurs’ (Figs. 3, 4, and 5). Before the CPE, rhynchosaurs and dicynodonts comprised 50–80% of individuals within well sampled faunas, whereas after the CPE they had dwindled to low abundance, and aetosaurs and sauropodomorph dinosaurs replaced them numerically, in some Norian faunas comprising 80–90% of individuals^13^. The TJE saw the end of aetosaurs, but sauropodomorphs continued to diversify and retained their ecological dominance as large herbivores, alongside the newly diversifying ornithischian dinosaurs.

The CPE did not cause the extinction of rhynchosaurs and dicynodonts but made them rare (Figs. 3 and 4). Rhynchosaurs went extinct in the early Norian^55^, whereas dicynodonts survived to the end of the Triassic, but at reduced diversity, abundance, and disparity^56,57^. This example shows the value of metrics of ecological abundance rather than species richness. Dicynodonts survived within wetter environments, even within dinosaur-dominated ecosystems^58^. Some of these latest taxa, such as *Lisowicia bojani* in Poland, even achieved huge body sizes that rivalled those of contemporaneous large sauropodomorphs^19,59^. The survival of kannemeyeriform dicynodonts might be because their typically large body sizes enabled them to explore wider geographic areas in search of suitable habitats. Nonetheless, they succumbed before the end of the Triassic alongside aetosaurs and cynognathian cynodonts^4^ (Fig. 3c). The end-Triassic saw widespread deforestation alongside a dramatic reorganisation of global floras that favoured ferns, at the expense of tropical flora^60,61^. Sparse floras dominated by ferns would have served as a poor food resource for large herbivores^62^ and therefore may be linked to the extinction of the aforementioned herbivore clades, which were largely low- to mid-level browsers.

We find that the largest episodes of morphospace expansion occur during the supposed recovery intervals of mass extinction events, with some surviving clades (particularly dinosaurs) showing much greater MO than before the extinction event (Fig. 5b). Morphospace expansion following the PTME occurs relatively quickly compared to the TJE, suggesting a relatively faster ecological recovery. Following the PTME and the loss of most species, total herbivore disparity and FFGs reached maximum levels in the Anisian, whereas following the TJE, the rebound in morphological diversity was modest, even by the end of the Early Jurassic. However, there is an edge effect here as we have not continued the analysis into the Middle Jurassic, and there may be sampling problems, as there are few well documented terrestrial tetrapod faunas in the Sinemurian and Pliensbachian. The inclusion of later dinosaur taxa and the overall diversification of dinosaurs in the Jurassic would likely yield a greater diversity of FFG in the later Jurassic than seen at the end of the Early Jurassic.

Furthermore, it is likely that the FFGs (light oral processors, shearing pulpers, and durophagous specialists) that disappeared within the Triassic would re-emerge as the climatic conditions stabilised from the end-Triassic event and terrestrial floras recovered^52,54^; the resurgence of floral diversity would likely have spurred new herbivorous diversification in both dinosaurs and mammals. The lost and depleted guilds identified here were likely restored as new dinosaurian and mammalian herbivores evolved through the later Mesozoic. Previous work highlights the prevalence of convergent evolution within dinosaurs^24^, and this is recognised here with repeated patterns of specialisation towards higher biting efficiency and greater oral processing in procolophonoids, rhynchosaurs, aetosaurs and ornithischians (Figs. 1 and 2). The prevalence of these patterns across quite phylogenetically distant clades emphasises that ecomorphs can disappear and reappear as conditions permit. This is further illustrated by the continuation of the prehension specialist FFG through the TJE with minimal change (Fig. 3b), despite the loss of its main constituent clade, the aetosaurs. The extinction of the aetosaurs in the TJE was offset by the emergence of heterodontosaurid ornithischians and likely later thyreophorans as the Jurassic progressed and they followed the common ‘herbivore adaptive pathway’ (Figs. 2 and 3c). Aetosaur-thyreophoran convergent evolution was not limited to jaw mechanics as ankylosaurs evolved similar armoured morphologies, and ecologies as large, quadrupedal, low-level feeders. However, these later thyreophorans developed more complex and powerful jaw mechanics^30^, allowing them to diverge from aetosaurs and exploit different niches as specialised herbivores.

Our study shows substantial ecological shifts occurred mostly at times of environmental instability, with only incremental development of ecospace during times of relative stability. This highlights a fluctuation between times of normal or ‘Red Queen’ evolution typified by adaptation to intrinsic pressures, punctuated by times of crisis or ‘Court Jester’ evolution, when large-scale extrinsic events provide the dominant selective pressures^7^. Our results confirm recent findings using model-based analyses that intrinsic, competitive interactions are the key to maintaining stasis within community assemblages through deep time^48,63^. Stasis is the norm, characterised by relatively stable climates and floras and honing of the adaptations of herbivores and slow expansion of morphospace occupation through biotic interaction. The environmental perturbations of the three global crises, all involving sharp global warming, extremes of humidity and aridity, and acid rain nearly but not quite killed off the dominant incumbent herbivores. The few survivors endured at the periphery of their former shape and function spaces, perhaps ecologically marginalised due to loss of food sources or because other surviving herbivores monopolised the newly prevalent vegetation. Episodes of instability mark a flip from dominance of competitive ability as the key driver of evolution to opportunism in perturbed times when the winners and losers might reflect entirely different selective advantages.

## Methods

### Taxonomic sampling and data collection

We compiled a list of all valid herbivorous tetrapod taxa from Early Triassic to Early Jurassic, using a published dataset^64^ and the latest literature to incorporate new taxa and taxonomic revisions. The stratigraphic ranges of these taxa (Supplementary Data 13) were updated to substage level following the designations of Benton et al.^64^. Absolute age assignments were based on the 2019 version of the International Chronostratigraphic Chart^65,66^. Assemblage data was gathered from Benton et al.^13^ for herbivore-rich early Mesozoic fossil localities and updated using published literature to include new taxa.

Our analysis was generally conducted at genus level to maintain a balance between availability of data and confidence in taxon diagnosis; in fact, most genera are monospecific. We generally used a single specimen per genus in this study, so we cannot account for varying levels of intraspecific variation; a true measure of total disparity would ideally include multiple specimens per taxon. Where intraspecific variation had been reported, we included more than one species for those genera, for example three species of the rhynchosaur *Hyperodapedon*: *H. gordoni*, *H. huxleyi* and *H. sanjuanensis*, from Europe, India, and South America, respectively, and four of *Lystrosaurus*: *L. hedini*, *L. maccaigi*, *L. murrayi*, and *L. robustus* from locations in China, South Africa and India. These were abundant and widespread taxa showing intrageneric shape variation. We also included all available cynognathian cynodonts, as some genera characterised as carnivores were found by isotopic analysis to have also fed on vegetation^67^, so omnivory may have been common within this group.

We compiled photographs and specimen drawings for 128 genera from the literature, taking care to exclude damaged, distorted, and juvenile material. These represent all taxa for which there is sufficient data for inclusion. The sample of 136 images includes 23 procolophonoid parareptiles, 22 dicynodont anomodonts, 17 cynognathian cynodonts, six tritylodont mammals, three bauriid therocephalians, seven ornithischian and 29 sauropodomorph dinosaurs, two silesaurids, eight aetosaurs, four pseudosuchians, and 15 non-archosaur archosauromorphs (Supplementary Data 12).

### Geometric and functional morphometrics

We used both geometric morphometric (GM) and functional morphometric (FM) methods to generate a detailed account of morphological and functional evolution in herbivorous tetrapod jaws. Using both methods allows for examination of changes in mandibular morphology alongside (clearly defined) biomechanical utility. GM methods capture the overall shape of the element of interest and FM methods capture biomechanical properties of the element and can thus give insight into function. These two methods can, but do not necessarily overlap in their results, since shape variation may be non-independent of some functional traits. Using both types of metric also allowed us to account for discrepancies between biomechanical and morphological patterns of disparity^68,69^. GM methods assess shape variation via user-defined landmarks and Cartesian coordinates, whereas FM methods use continuous functional measurements such as mechanical advantage (MA) and aspect ratio, which reflect biting efficiency and jaw robusticity, respectively^70,71^. We used both Procrustes aligned landmark data and standardised functional measurement data (SFMD) that were selected from previous studies of tetrapod feeding morphology^69–72^.

### Shape data

Herbivorous tetrapods encompass a wide range of mandible morphologies making it difficult to identify more than a small number of homologous landmark points. We opted for a relaxed landmarking regime, in which we used four fixed landmarks connected with four semi-landmarked curves comprising of 55 semi-landmarks in total (Supplementary Fig. 1). Hence, our landmarking regime focuses on overall shape (type 2 landmarking), rather than contacts between bones of the mandible (type 1 landmarking). Type 1 landmarking was impractical as contacts were not clearly visible across our specimens due to the aforementioned shape variability, and homologies were hard to ascertain because of the wide phylogenetic range of the included genera.

Images were digitally landmarked using tpsDig2^73^, with fixed landmarks placed at homologous points on each mandible and semi-landmarks equally spaced along curves between the fixed landmarks. We used tpsUtil^74^ to enable semi-landmarks to slide along their respective curves during the Procrustes transformation using the chord–min *d*^2^ sliding method that allows each semi-landmark to slide along a chord between the two adjacent landmarks. Procrustes transformation was carried out using tpsRelW^75^ to remove the effects of mandible size and orientation from the landmark data and to generate aligned coordinates (Supplementary Data 1).

### Functional data

We collected data for eight functional characters using measurements taken from our mandible images (Supplementary Table 1, Supplementary Fig. 2, and Supplementary Data 2). These measurements, taken with ImageJ^76^, were chosen specifically to capture biomechanically relevant components of mandible shape, especially areas of muscle attachment, articulation, and overall mandible shape that have a known relationship to feeding ecology^69–72^. Variables that are not closely associated with biomechanical properties were purposefully excluded. (See Supplementary Fig. 2. For full details of how these measurements were collected.)

### Principal component analysis

To identify the major axes of variation, the shape-aligned coordinate data and functional measurement matrix were subjected to principal component analyses (PCAs). A PCA transforms total variation into a matrix of independent variables (PC axes) (Supplementary Data 3 and 4). For the PCA analyses, we used packages in R^77^ (R Core Team, 2018), including geomorph^78^ for the aligned coordinate data and FactoMineR^79^, to centre and apply a z-transformation to the functional measurements prior to a PCA following established protocols^24,70^. An alternative PCA was carried out using an alternative data standardisation to assess the robusticity of the PCA results reported above (see supplement). The resulting morphospaces are different (Supplementary Fig. 5), likely as a result of the different treatment of the underlying trait data, but the overall results remain consistent across all methods and do not change the broader findings presented here.

The first and second principal components were used to plot morphospace occupation, with these components amounting to 32% and 14% of total shape variation, and 42% and 16% of total functional variation, respectively, constituting the maximum morphological variation within two components. Functional character contour plots were generated using the akima package^80^, with linear interpolation of functional and PC data for all taxa generating functional data for all areas of occupied morphospace.

### Cluster analyses

Our goal was to empirically identify distinct clusters that represent differentiated dietary niches as can be recognised in fossils with as little ambiguity as possible. To accomplish this, we employed several different clustering analyses, each with its own analytical strategy, and looked for clusters that were common to them all with the intention of defining groups that can be easily recovered no matter what method is employed. We used the SFMD to define functional feeding groups (FFGs) because these traits have known links to feeding ecology and diet in extant taxa^81^, hence allowing us to interpret differences in disparity from an eco-functional perspective rather than more ambiguous comparisons of shape. It should be noted that our functional characters include some that are based on functionally important aspects of shape, which could lead to some similarities if the cluster analyses were applied to shape rather than functional data. However, landmark data encapsulates a greater level of shape detail and disregards aspects such as muscle attachment positions, and so we expect a decoupling between the results of cluster analyses run using either the functional or shape landmark as seen in other comparative studies of form and function^69^.

Boundaries between dietary niches, particularly when dealing with extinct taxa, are increasingly ambiguous beyond broader groupings such as herbivore or carnivore, and generalist or specialist. Realised niches often vary due to factors such as the conspecifics present and available resources^82,83^. As such, we employ hierarchical and two partition clustering methods: *K*-means and partitioning around medioids (PAM)^84^. These methods group taxa into clearly defined ‘hard’ clusters using machine-learning algorithms that require minimal prior input, thus bolstering the objectivity of resulting cluster groups. All analyses are unsupervised and use different clustering algorithms, which complement each other when used in combination. Agglomerative hierarchical clustering is a distance-based method that uses a ‘bottom-up’ approach to assign taxa to progressively larger groupings, whereas *K*-means and PAM are partition methods that use randomly selected centroids/medoids to assemble optimal cluster configurations based on cluster cohesion and separation^84^. *K*-means clustering focuses on minimising the sum of squared Euclidean distances and uses artificial centroids, whilst PAM tries to minimise the sum of general pairwise dissimilarities and uses real data-points (medoids) as the centroids and is also considered more robust to outliers and noise within the data^85^. These methods complement each other as *K*-means may better identify the core taxa in each FFG, but struggle with classifying peripheral taxa, but PAM can recover irregular cluster configurations, which may more accurately reflect niche-spaces within the overall morphospace. The hierarchical cluster analysis forms a tree based on phenetic similarities, assuming a parsimonious regime of trait evolution.

The three separate analyses were applied to a Euclidean distance matrix generated using the SFMD. Using Euclidean distances is an appropriate choice given our continuous multivariate dataset and the aim to use the magnitude of differences between taxa to determine separate groups, as well as enabling use of subsequent partition clustering methods. The hierarchical analysis was carried out first to explore the clustering present within our taxa as the agglomerative process enables identification of the clusters and subclusters present, as well as the degree of separation between these groupings. These results inform the subsequent *K*-means and PAM analyses, which both require a user-defined range of cluster combinations to test^85^. The cluster analyses were run in R using the ‘eclust’ function from the factoextra package^86^, with the partition methods identifying the optimal number of clusters from within our defined cluster (*K*) range (4–10) using gap statistic values generated from 2000 bootstrap cycles. The hierarchical analysis was also rerun using the defined *K* range to generate clear cluster classifications. The results from these three cluster analyses (Supplementary Data 5) were validated using the ‘cluster.stats’ function from the fpc R package^87^. Resulting cluster ‘silhouette’ metrics^84^ (Supplementary Table 5) show that all three cluster methods performed fairly equally, so all three cluster analysis results were used to generate composite groups to act as our FFGs, based on patterns of consensus in the distribution of taxa across clusters (Supplementary Data 5). Where possible, we used lower-level taxonomic groupings to increase FFG assignment accuracy. FFGs were assigned to clades based on which groups held the majority of a clade’s taxa. This approach enabled us to better compare taxa in different assemblages in the later assessment of potential competition. This coarse classification scheme may conceal the true levels of feeding diversity present, but because many assemblages feature taxa not included in this analysis, we felt that this cautious approach would ensure a more robust assessment of potential competition. Further study utilising new and alternative aspects of feeding anatomy (such as dentition) may enable higher resolution classification of feeding diversity in future.

A disproportionately large number of (predominantly sauropsid) taxa were recovered within a single cluster group, and while we termed this group ‘ingestion generalists’, we felt this grouping provided little diagnostic use as an FFG in our investigations of potential competition. Therefore, we re-ran the above cluster procedures using only the ingestion generalist taxa in an attempt to recover more details of potential clade-level competition, and we then identified three FFsGs (basal generalists, tough generalists, and light oral processors).

We further tested the robusticity of our FFGs by running the above cluster analyses and FFG construction using data subject to alternative standardisation (see supplement). The resulting FFGS show some classification differences within the sauropsids and a shift in the boundary of the ingestion generalists and prehension specialists (Supplementary Data 15–16 and Supplementary Fig. 6). These differences reflect the strong levels of morphological conservatism within the sauropsids and key changes in the relative importance of our functional characters. However, the core assortment of taxa in each FFG largely remains and the alternative FFG results do not change the conclusions presented here.

### Calculations of disparity through time

Disparity is a measure of morphological diversity that is calculated using the volume and extent of morphospace occupation. To explore patterns of shape and functional disparity, we calculated within‐time‐bin mean pairwise distances (MPD) (henceforth variances) using a Euclidean distance matrix generated from the aligned landmark data. MPD is a fairly conservative measure of disparity and although it may not fully illustrate the extent of occupied morphospace, it is resistant to sample size inconsistencies and an effective metric for measuring relative changes in morphospace^88^, which is of key interest to this study. We used 1000 cycles of bootstrapping to provide 95% confidence intervals. Our plots were generated in R using the calibrate package^89^. ‘Morphospace packing’ (increasing density within morphospace) has been shown to reduce disparity by lowering the average dissimilarity, despite the overall morphospace area/volume remaining stable^90,91^. Consequently, we plotted MPD alongside substage level, time-slices of morphospace in order to avoid misinterpreting the disparity results.

To quantitatively assess the significance of changes in morphospace through time, a one-way non-parametric analysis of variance (NPMANOVA) using a Euclidean similarity index was applied at epoch and stage level in PAST^92^ (version 3.24) to the aligned landmark shape data and functional SFMD. Bonferroni corrections were also applied owing to the multiple comparisons carried out.

### Calculations of divergence through time

To quantify the contrasting eco-evolutionary trajectories of the three main clades analysed here (Archosauromorpha, Therapsida and Parareptilia), we calculated the mean shape and functionality for each clade at stage level using the aligned landmark shape data and functional SFMD. The mean values were subsequently used to generate Euclidean distance matrixes for the shape and functional data, respectively.

### Reporting summary

Further information on research design is available in the Nature Research Reporting Summary linked to this article.

## Supplementary information

Supplementary Information

Description of Additional Supplementary Files

Supplementary Data 1-10

Supplementary Data 11-16

Reporting Summary

## Data Availability

The authors declare that all the data directly supporting the results of this study and underlying all figures are included within the supplementary data files linked to this paper and are also available at 10.5061/dryad.0cfxpnw24.

## Code availability

The authors have included R scripts to perform the analyses used in this study within Supplementary Note 2.
