# Author Correction: Niche partitioning shaped herbivore macroevolution through the early Mesozoic

**DOI:** 10.1038/s41467-021-24593-9

**Published:** 2021-07-22

**Authors:** Suresh A. Singh, Armin Elsler, Thomas L. Stubbs, Russell Bond, Emily J. Rayfield, Michael J. Benton

**Affiliations:** School of Earth Sciences, University of Bristol, Bristol, UK

**Keywords:** Palaeoecology, Palaeontology

Correction to: *Nature Communications* 10.1038/s41467-021-23169-x, published online 14 May 2021.

The original version of this Article contained an error in Fig. 1, in which several of the silhouettes did not appear in panel j. The original version of Fig. 1 is


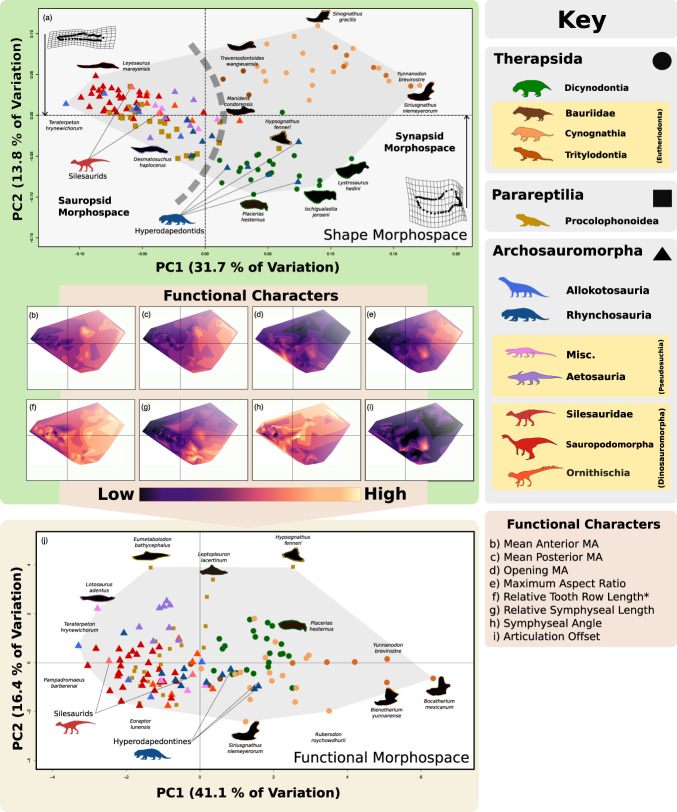


and the corrected version is


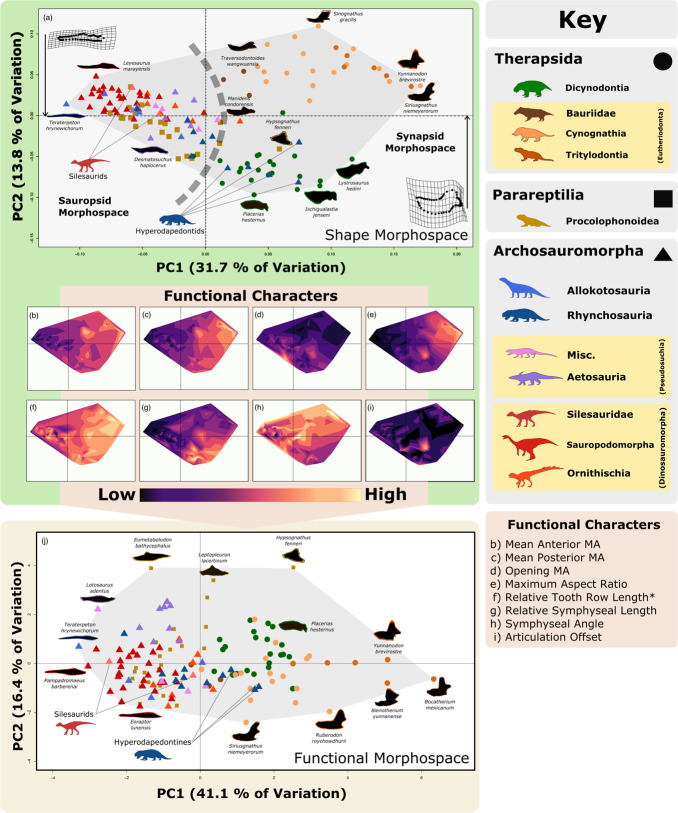


This has been corrected in both the PDF and HTML versions of the Article.

