# Supplementary material for: Niche partitioning shaped herbivore macroevolution through the early Mesozoic
Source: Nat Commun. 2021 May 14;12:2796. doi: 10.1038/s41467-021-23169-x (PMC8121902; doi:10.1038/s41467-021-23169-x)
Supplement: Supplementary file 1 — Supplementary Information [file 41467_2021_23169_MOESM1_ESM.pdf]

Supplementary Information

Niche partitioning shaped herbivore macroevolution through the early Mesozoic

Suresh A. Singh[1]*, Armin Elsler[1], Thomas L. Stubbs[1], Russell Bond[1], Emily Rayfield[1] & Michael J. Benton[1].

1. School of Earth Sciences, University of Bristol, Life Sciences Building, Tyndall Avenue, Bristol, BS8 1TQ, UK. *e-mail: ss1314@bristol.ac.uk

Contents:

Supplementary Note 1..3

Alternative data transformation methods and results

Supplementary Table 1..5

Functional character descriptions and functional utility to feeding behaviour.

Supplementary Table 2..7

Character loadings for functional principal component (fPC) scores in main text.

Supplementary Table 3..8

Character loadings for functional principal component (fPC) scores in supplement.

Supplementary Table 4..8

Symmetric Procrustes analysis results.

Supplementary Table 5..9

Internal validation statistics for different cluster configurations.

Supplementary Table 6..9

External validation statistics for different cluster configurations.

Supplementary Table 7..10

Feeding functional group conflicts within early Mesozoic assemblages.

Supplementary Table 8..11

Total herbivore shape and functional disparity at stage level.

Supplementary Table 9..12

Archosauromorph shape and functional disparity at stage level.

Supplementary Table 10…………………………………………………………………………………12
Parareptile shape and functional disparity at stage level.

Supplementary Table 11…………………………………………………………………………………13
Therapsid shape and functional disparity at stage level.

Supplementary Table 12...…………………………………………………………………………...….14
Centroid distances through time per clade.

Supplementary Table 13...…………………………………………………………………………...….15
Shape and functional distances between clade centroids through time.

Supplementary Table 14…………………………………………………………………………………16
PERMANOVA results for statistical significance of shape and functional morphospace changes through stage and epoch transitions.

Supplementary Figure 1……………………………………………………………………………...….17
Geometric morphometric landmarking regime.

Supplementary Figure 2……………………………………………………………………………...….18
Functional character measurements guide.

Supplementary Figure 3……………………………………………………………………………....…19
Feeding Functional Group Characters (– main groups only).

Supplementary Figure 4……………………………………………………………………………...….20
Shape morphospaces using combinations of the first three principal components.

Supplementary Figure 5……………………………………………………………………………...….21
Functional morphospace results from PCA only z and z and logit transformed data.

Supplementary Figure 6……………………………………………………………………………...….22
Logit functional feeding group classifications and comparison

Supplementary Note 2: R Code…………………………………………………………………...….23

Supplementary References……………………………...…………………………………………..26

Supplementary Note 1

Alternative data transformation methods. In order to assess trophic macroevolution using multivariate data, we applied principal component analyses (PCAs) to the shape-aligned coordinate data and functional measurement matrix. The functional dimensional scaling was executed using multiple procedures to offset the impacts of potential violations of the data assumptions in a PCA. A z-standardisation to the continuous character data prior to running the PCA was applied following the procedures of many previous studies[1,3–6]. We also explored the impact of using an additional logit standardisation on the proportional ratio characters (all characters barring the symphyseal angle) prior to the z transformation using the gtools R package[7]. This was done to enable our data to better fulfil the linear assumptions of subsequent analyses and test the potential impacts of non-linearity in our data, which is often an issue of ratio data[8]. As a monotonic function, the logit transformation has become a favoured option for linearising proportional data, particularly when variance stabilisation is no longer a prime concern as is the case with non-binomial ratios [9]. The transformation proved inapplicable to two tritylodont taxa (*Bienotherium* and *Bocatherium*) due to their exceedingly high posterior mechanical advantage values, meaning this character was automatically adjusted to the mean values for these taxa in a PCA. However, we used the 'fill.missing' function from the nbpMatching package[10] to impute the missing values using the greatest correlation with the best linear combination of the other characters.

The resulting morphospaces show subtle differences (Supplementary Fig. 4 and 5), likely due to the different algorithms employed by each method. Comparison of the different results shows that the broad patterns of different groupings and associations tend to remain constant, and an additional Procrustes correlation analysis conducted using the vegan r package[11] found strong correlation between the z and logit transformed PCA scores (Supplementary Table 4). There are some slight differences on the lower PC axes that result from emphasis being placed on different characters (Supplementary Table 1). This nonetheless alters the placement of some taxa, mainly the aetosaurs and the leptopleuronid procolophonids.

Alternative cluster analysis results. Subtle differences in the PCA results led us to further test the robusticity of the functional feeding group (FFG) assignments by rerunning the cluster analyses using the logit transformed data. The resulting FFGs show close similarities to those derived from the z transformed data (Supplementary Figure 6). However, there are some prominent differences within the sauropsids as the logit cluster results (Supplementary Data 15; Supplementary Data 16) show that the tough generalist and light oral processor functional feeding subgroups remain largely intact but are

now grouped with prehension specialists instead of the ingestion generalists. Additionally, the unique durophagous specialist FFG populated exclusively by the leptopleuronid procolophonids are now grouped with the shearing pulper group. These changes reflect the impact of the logit transformation on the relative weighting of the functional characters, with the relative articulation offset character in particular losing discriminatory power and being reflected in lower PC axes (Supplementary Table 3). It should be noted the relative articulation offset was a character that distinguished the durophagous specialists in the main (z transformed) results (Fig. 2). The differences evidently stem from the alternative treatment of the data, but we also suggest that this is a result of the strong sauropsid morpho-functional conservatism as highlighted in the discussion. The general indistinctiveness of niche boundaries combined with high levels of similarity between sauropsid taxa means that changes to classifications for taxa on the peripherals of cluster 'cores' is not unexpected. Indeed, we notice that the cluster cores remain largely intact, affirming the general robusticity of our FFGs.

The slight differences noted here should not be overlooked, but as the fundamental relationships that are key to our later competition analyses remain relatively constant, we retain the 'z transformed only' PCA results as they offer greater discrimination between groups and enable more coherent comparison with previous studies of some of the amniote groups presented here[1,3]. The logit results do not dispute the core findings of this study which identify stronger support for potential competition between sauropsids, particularly the archosaurs, rather than between sauropsids and synapsids in the Triassic.

Supplementary Table 1. Functional character descriptions and functional utility to feeding behaviour. Abbreviations: MA= Mechanical advantage.

Functional Character	Description	Jaw Functionality
1. Mean Anterior Mechanical Advantage	A measure of biting efficiency at the anterior of the mandible[12]. This is the ratio of the inlever to the outlever, using the distance from the jaw joint to the anterior-most tip of the toothrow/dentary as the outlever. The distance from the jaw adductor muscle attachment to the jaw joint represents the inlever. This ratio of inlever to outlever gives the lowest possible value of MA.	Mandibular function can be described using lever mechanics with the jaw acting as a third-order lever system[12,13]. The adductor musculature acts as the input force, the craniomandibular joint acts as the fulcrum and the output force is exerted along the toothrow/shearing surface.<br>Herbivores often exhibit higher MA values than faunivores[14].<br>Levers are measured from the craniomandibular joint/jaw articulation.<br>Taxa with low MA exhibit weak and rapid bites[5,15], whilst taxa with a strong bite force have a high MA.<br>Mean values generated from the MA values for different adductor muscle groups to account for differences in jaw musculature between herbivorous taxa (Supplementary Fig 1).
2. Mean Posterior Mechanical Advantage	A measure of biting efficiency at the posterior of the mandible[12]. This is the ratio of the inlever to the outlever, using the distance from the jaw joint to the posterior-most point of the toothrow/dentary as the outlever. The distance from the jaw adductor muscle attachment to the jaw joint represents the inlever. This ratio of inlever to outlever gives the highest possible value of MA.	
3. Opening Mechanical Advantage	A measure of biting velocity[12]. This is the ratio of the maximum inlever to the maximum outlever, using the distance from the jaw joint to the posterior-most point of the mandible/retroarticular process for the inlever, and using the distance from the jaw joint to the posterior-most point of the toothrow/dentary as the outlever. Opening MA is linked to feeding patterns and prey selection[5,16].	

4. Relative Maximum Aspect Ratio	A proxy for the second moment of area, previously used in 2D analyses of jaw mechanics[4,5]. Generated by dividing the maximum depth of the mandible by its total length.	Most mandibles primarily experience dorsoventral stress during feeding function therefore the aspect ratio represents a measure of flexural stiffness[6].
5. Relative Toothrow Length	A measure of relative length of the dentition and its purported importance in trophic behaviour[2]. Generated by dividing the length of the toothrow/shearing surface by the total length of the mandible.	A longer toothrow enables a greater range of MA along the jaw and likely increased use of the dentition in jaw functionality (either for food ingestion or processing/mastication). Herbivores tend to show relatively shortened toothrows compared to faunivores and omnivores[17].
6. Relative Symphysis Length	A measure of symphyseal robusticity generated by dividing the length of the symphysis by the total length of the mandible.	The symphysis is subject to significant bending, shear, and torsional stress during biting action and so is highly related to transmission of muscle and biting force and feeding ecology and overall jaw mechanics[18,19].
7. Symphyseal Angle	The symphyseal angle is measured between the ventral jaw line and a line parallel to the long axis of the mandibular symphysis.	The angle of the symphysis affects symphyseal resistance to the bending, shear, and torsional stresses that occur during the bite cycle[18]. It is known to affect food processing in modern herbivorous rhynchocephalians[19] and is of major importance in the mechanical response of modern crocodylians to biting, twisting, and shaking[20,21].
8. Relative Offset of Articulation	The articulation offset is measured as the length of the line perpendicular to the tangent of the mandibular toothrow (extrapolated from the anterior and posterior ends of the toothrow to account for jaw curvature) which intersects the articular joint[4,6]. This value is then divided by the total jaw length.	An offset between the toothrow and jaw articulation affects dental occlusion and leverage of the jaw musculature[22]. A small articulation offset indicates 'scissor-like' occlusion, which is typical of carnivorous taxa. Herbivores generally exhibit greater toothrow-articular offset as this enables simultaneous occlusion along the entirety of the toothrow, supporting gripping & crushing actions[23].

Supplementary Table 2. Character loadings to functional principal component (fPC) scores using Z-transformed data. (Results presented in main text.) Abbreviations: MA, Mechanical advantage.

Functional Characters	fPC 1	fPC 2	fPC 3	fPC 4	fPC 5	fPC 6	fPC 7	fPC 8
Mean Anterior MA	0.4934625	0.24667339	-0.0900206	0.00786307	-0.1050386	-0.3682956	-0.4319062	0.59520067
Mean Posterior MA	0.5254681	-0.0619115	0.06039547	-0.0601783	0.2154931	-0.0632664	-0.4132983	-0.7010889
Opening MA	-0.1631619	0.5947072	0.14454208	-0.2878902	0.7146802	0.01233845	0.02485324	0.06626129
Max Aspect Ratio	0.495494	0.08422196	-0.0582864	0.21013936	0.1223364	-0.2877307	0.77419792	-0.0519512
Relative Toothrow Length	0.2882966	-0.5297162	0.33770575	0.07496055	0.4259769	0.43923177	-0.0005719	0.37714757
Relative Symphyseal Length	0.3340768	0.23554854	0.06918551	-0.622936	-0.4008042	0.49565115	0.18327337	0.0130569
Symphyseal Angle	0.1052323	0.16547329	-0.765179	0.31068997	0.158592	0.49986206	-0.065635	0.01400572
Quadrate Articular Offset	0.0494548	0.4594403	0.50953734	0.61569542	-0.2225842	0.29825801	-0.0688771	-0.0671866

Supplementary Table 3. Character loadings to functional principal component (fPC) scores using logit and Z-transformed data. Abbreviations: MA, Mechanical advantage. *Character not subject to logit transformation.

Functional Characters	fPC 1	fPC 2	fPC 3	fPC 4	fPC 5	fPC 6	fPC 7	fPC 8
Mean Anterior MA	0.49503534	0.23122617	0.10536859	0.04964019	-0.1273287	-0.3363787	-0.1565343	0.73078148
Mean Posterior MA	0.49693454	-0.1216105	0.07959073	-0.1474894	0.18112881	-0.1646706	-0.6462706	-0.4822753
Opening MA	-0.1806332	0.32970026	0.53062276	-0.5059771	0.55308876	-0.0403764	0.09063646	0.07310027
Max Aspect Ratio	0.49570689	0.05747547	0.00678971	0.12181526	0.13688021	-0.3172677	0.71115436	-0.3330926
Relative Toothrow Length	0.26850726	-0.6430047	-0.1077064	-0.0763911	0.47019064	0.39168346	0.11757984	0.32968526
Relative Symphyseal Length	0.3813682	0.21990249	0.22890568	-0.2835418	-0.4432088	0.67654042	0.11067584	-0.0837695
Symphyseal Angle*	0.10931081	0.59506341	-0.4519173	0.31625262	0.44803486	0.34126223	-0.1116049	-0.0074124
Quadrate Articular Offset	-0.0124778	-0.0618631	0.657821	0.72019698	0.08993274	0.1728038	-0.0731168	-0.0361939

Supplementary Table 4. Symmetric Procrustes analysis illustrating the degree of correlation between the functional principal component scores generated using alternate data transformation regimes: using a Z-transformation and using both logit and Z-transformations.

Symmetric Procrustes Analysis Results	
Procrustes Sum of Squares	0.05407
Correlation in a symmetric Procrustes rotation	0.9726
Significance	0.0001

Supplementary Table 5. Internal validation statistics for different cluster configurations**.** Abbreviations: PAM, partition around medoids. WSS, within cluster sum of squares. MDC, mean distance from cluster centroid. MDB, mean distance between cluster centroids. pF Value, pseudo F value.

Clustering Method	WSS	MDC	MDB	pF Value	Dunn Index
Hierarchical	528.7776	2.667516	4.158011	1.558758	0.1247351
K-means	495.668	2.605416	4.16443	1.598375	0.1466154
PAM	543.0932	2.683238	4.062733	1.514116	0.06729752

Supplementary Table 6. External validation statistics for different cluster configurations**.** Abbreviations: PAM, partition around medoids. WSS, within cluster sum of squares. MDC, mean distance from cluster centroid. MDB, mean distance between cluster centroids. pF Value, pseudo F value.

Phylogenetic Groups	Corrected Rand Index		Meila's VI Index	
	Broad Clades	Higher Resolution Clades	Broad Clades	Higher Resolution Clades
Hierarchical	0.2871463	0.08786818	1.947933	2.703176
K-means	0.3190135	0.1046528	1.824302	2.567004
PAM	0.341187	0.1223362	1.939353	2.603949

Supplementary Table 7. Feeding functional group conflicts within early Mesozoic assemblages. The total proportions herbivore and herbivorous dinosaur species also shown. Abbreviations: IG, Ingestion generalists. Clades: B. PrcD, Basal Procolophonidae. B. RhyncD, Basal Rhynchosauridae. ProcN, Procolophoninae. RhyncD, Rhynchosauridae. Hyp. RhyncD, Hyperodapedontine Rhynchosauridae. Lept. PrcD, Leptopleuronine Procolophonidae. B. SauropodM, Basal Sauropodomorpha. B. Thyreophora, Basal Thyreophora. Misc. Psd, Miscellaneous Pseudosuchia. Assemblage data from the Early Tetrapod Dataset (ETD) by Benton et al.[24].

Assemblages	Potential FFG Conflicts	Groups Involved	Conflict FFG
Lystrosaurus AZ	3	Procolophonoidea	IG - Tough Generalists
Cynognathus AZ (B)	3	Rhynchosaurs, Procolophonidae, and Bauriidae	IG - Tough Generalists, IG – Light Oral Processors, Prehension Specialists
Lower Ehrmaying	0		
Upper Ehrmaying	0		
Yerrapalli	0		
Donguz	0		
Lifua	0		
Manda	0		
Lower Ntawere	0		
Isalo II	0		
Lower Santa Maria	1	Dicynodonts and Hyperodapedontine rhynchosaurs	Shearing Pulpers
Lower Ischigualasto	4	Silesauridae and Aetosauria and Poposauridae	Prehension Specialists
		Dicynodonts and Hyperodapedontine rhynchosaurs	Shearing Pulpers
Argana	0		
Lossiemouth	0		
Colorado City	1	Trilophosaurs and Aetosaurs	Prehension Specialists
Santa Maria U	0		
Tecovas	1	Trilophosaurs and Aetosaurs	Prehension Specialists
Caturrita	1	Sauropodomorpha	IG - Basal Generalists
Lower Elliot	2	Sauropodomorpha	IG - Basal Generalists
		Thyreophoran Ornithischians and Sauropodiformes	IG - Tough Generalists
Los Colorados U	1	Sauropodomorphs	IG - Basal Generalists
Cooper Canyon	1	Shuvosaurids and Aetosaurs	Prehension Specialists
Knollenmergel	0		
Redonda	0		
Zhangjiawa	1	Sauropodomorpha and Pseudosuchia	Prehension Specialists
Kayenta	1	Misc. Pseudosuchia and Tritylodontia	Heavy Oral Processors
Upper Elliot	4	Thyreophoran Ornithischians, Sauropodiformes and Sauropods	IG - Tough Generalists
		Ornithischians and Sauropods	Prehension Specialists

Supplementary Table 8. Results for herbivore shape and functional disparity at stage level with minimum and maximum bounds for 95% confidence intervals. Abbreviations: L., Lower. M., Middle. Max, Maximum. Min, Minimum. MPD, Mean pairwise distance. U., Upper.

	Timebin	Shape			Function		
		MPD	MPD min	MPD max	MPD	MPD min	MPD max
Total herbivore disparity	Induan	0.1445887	0.13483597	0.1542025	3.100405	2.873077	3.326893
	Olenekian	0.1432726	0.13699571	0.1495954	3.429627	3.194541	3.672072
	Anisian	0.1579298	0.15496873	0.1608701	3.466344	3.376638	3.55972
	Ladinian	0.162579	0.15765787	0.1674503	3.752859	3.536768	3.969487
	L. Carnian	0.1652017	0.15989481	0.1706082	3.538404	3.289624	3.785865
	U. Carnian	0.1750951	0.16897517	0.1813733	4.04509	3.905203	4.183961
	L. Norian	0.1668659	0.15825504	0.1756525	3.488175	3.33425	3.640399
	M. Norian	0.1235526	0.11549975	0.131735	3.309642	3.10023	3.516843
	U. Norian	0.1447827	0.13058011	0.1593925	4.068642	3.690119	4.464911
	Rhaetian	0.183866	0.15975648	0.2073251	4.598277	4.053449	5.15978
	Hettangian	0.1261408	0.11740697	0.1352225	3.3409	3.094312	3.585143
	Sinemurian	0.1447199	0.13545447	0.1540862	3.741877	3.509254	3.982271
	Pliensbachian	0.1880424	0.17090689	0.2064568	4.863254	4.055635	5.700277
	Toarcian	0.1064164	0.08799137	0.1248414	2.932972	2.387499	3.478445

Supplementary Table 9. Results for archosauromorph shape and functional disparity at stage level with minimum and maximum bounds for 95% confidence intervals. Abbreviations: L., Lower. M., Middle. Max, Maximum. Min, Minimum. MPD, Mean pairwise distance. U., Upper.

	Timebin	Shape			Function		
		MPD	MPD min	MPD max	MPD	MPD min	MPD max
Archosauromorpha disparity	Induan	NA	NA	NA	NA	NA	NA
	Olenekian	NA	NA	NA	NA	NA	NA
	Anisian	0.10474423	0.09228994	0.1175903	2.307067	2.015081	2.565871
	Ladinian	0.1278479	0.1278479	0.1278479	4.426436	4.426436	4.426436
	L. Carnian	0.17397138	0.1560309	0.19191187	5.197552	4.563563	5.831541
	U. Carnian	0.12729403	0.1196138	0.13517212	3.54249	3.355211	3.72607
	L. Norian	0.09847115	0.09385611	0.10321581	2.747459	2.580191	2.920414
	M. Norian	0.09658072	0.09307374	0.10005029	2.8028	2.615463	2.984609
	U. Norian	0.10157567	0.09590351	0.10746544	3.079998	2.822621	3.342785
	Rhaetian	0.09058495	0.0762065	0.10703437	2.636028	2.368081	2.894308
	Hettangian	0.09746722	0.09331604	0.10178418	2.710316	2.523729	2.899712
	Sinemurian	0.09116253	0.08783565	0.09440833	2.579776	2.425916	2.737153
	Pliensbachian	0.16169554	0.16169554	0.16169554	5.004178	5.004178	5.004178
	Toarcian	0.10641641	0.08799137	0.12484144	2.932972	2.387499	3.478445

Supplementary Table 10. Results for parareptile shape and functional disparity at stage level with minimum and maximum bounds for 95% confidence intervals. Abbreviations: L., Lower. M., Middle. Max, Maximum. Min, Minimum. MPD, Mean pairwise distance. U., Upper.

	Timebin	Shape			Function		
		MPD	MPD min	MPD max	MPD	MPD min	MPD max
Parareptilia disparity	Induan	0.09149552	0.07986558	0.1035187	1.914165	1.702471	2.127557
	Olenekian	0.12351262	0.11468565	0.1320419	3.093589	2.756892	3.446809
	Anisian	0.13327885	0.12129245	0.146882	3.445285	2.796384	4.119149
	Ladinian	0.19631361	0.19631361	0.1963136	5.265504	5.265504	5.265504
	L. Carnian	NA	NA	NA	NA	NA	NA
	U. Carnian	NA	NA	NA	NA	NA	NA
	L. Norian	NA	NA	NA	NA	NA	NA
	M. Norian	NA	NA	NA	NA	NA	NA
	U. Norian	NA	NA	NA	NA	NA	NA
	Rhaetian	NA	NA	NA	NA	NA	NA
	Hettangian	NA	NA	NA	NA	NA	NA
	Sinemurian	NA	NA	NA	NA	NA	NA
	Pliensbachian	NA	NA	NA	NA	NA	NA
	Toarcian	NA	NA	NA	NA	NA	NA

Supplementary Table 11. Results for therapsid shape and functional disparity at stage level with minimum and maximum bounds for 95% confidence intervals. Abbreviations: L., Lower. M., Middle. Max, Maximum. Min, Minimum. MPD, Mean pairwise distance. U., Upper.

	Timebin	Shape			Function		
		MPD	MPD min	MPD max	MPD	MPD min	MPD max
Therapsida disparity	Induan	0.1338565	0.1221877	0.145506	2.278369	2.065287	2.499334
	Olenekian	0.1344112	0.120528	0.1469067	2.716522	2.157714	3.25092
	Anisian	0.1530128	0.1481407	0.1577795	3.019929	2.916729	3.12159
	Ladinian	0.1489864	0.1430042	0.1549179	2.998761	2.77864	3.21537
	L. Carnian	0.1522754	0.146442	0.1583021	2.750761	2.559374	2.944616
	U. Carnian	0.2089568	0.194219	0.2222803	3.309207	2.914151	3.714083
	L. Norian	0.2178604	0.2035501	0.2305253	3.497063	3.067216	3.894637
	M. Norian	0.1589451	0.1589451	0.1589451	2.987786	2.987786	2.987786
	U. Norian	NA	NA	NA	NA	NA	NA
	Rhaetian	0.2031484	0.2031484	0.2031484	3.739695	3.739695	3.739695
	Hettangian	0.0949083	0.0949083	0.0949083	2.511281	2.511281	2.511281
	Sinemurian	0.1710371	0.142037	0.198981	2.987366	2.677312	3.332465
	Pliensbachian	0.1558767	0.1479013	0.1638522	3.287281	2.687763	3.8868
	Toarcian	NA	NA	NA	NA	NA	NA

Supplementary Table 12. Shape and functional differences through time by clade. Euclidean distances of taxa in successive timebins. The mean change between timebins is highlighted in bold, with greater than average changes in italic. Abbreviation: ANS, Anisian. CRN, Carnian. HET, Hettangian. IND, Induan. LAD, Ladinian. M, Middle. NOR, Norian. OLE, Olenekian. PLB, Pliensbachian. RHT, Rhaetian. SIN, Sinemurian. TOA, Toarcian.

Raw distances	PR GM Distance	PR FM Distance	ThP GM Distance	ThP FM Distance	Arch GM Distance	Arch FM Distance
IND to OLE	0.037588717	0.765074367	0.09602917	2.550595194	NA	NA
OLE to ANS	0.040774895	1.159737606	0.046512798	1.194520406	NA	NA
ANS to LAD	0.081554452	2.11375207	0.037871321	0.699428297	0.054723111	1.304334986
LAD to L. CRN	0.132998282	2.855256367	0.037419148	0.710375579	0.060287797	1.726533898
L. CRN to U. CRN	NA	NA	0.082236526	1.205805001	0.051580074	1.365566866
U. CRN to L. NOR	NA	NA	0.027494766	0.553003559	0.031315572	0.616074704
L. NOR to M. NOR	NA	NA	0.097036481	2.221965681	0.013964427	0.529565097
M. NOR to U. NOR	NA	NA	0.079472209	1.499415491	0.012741677	0.23603122
U. NOR to RHT	NA	NA	0.101573793	1.876759861	0.052903971	1.655405751
RHT to HET	NA	NA	0.100056957	2.219552363	0.043658529	0.835106611
HET to SIN	NA	NA	0.05032122	0.890317593	0.020617797	0.619080702
SIN to PLB	NA	NA	0.100408418	1.020866782	0.056729342	1.38183536
PLB to TOA	NA	NA	NA	NA	0.078989447	1.489050518
Mean distance	0.073229086	1.723455102	0.066336469	1.248363408	0.042809224	1.05013389

Supplementary Table 13. Shape and functional distances between clade centroids through time. Abbreviations: ArchM, Archosauromorpha. L., Lower. M., Middle. PR, Parareptile. ThP, Therapsida. U., Upper.

Timebin	Shape			Function		
	PR-ArchM	PR-ThP	ThP-ArchM	PR-ArchM	PR-ThP	ThP-ArchmM
Induan	NA	0.15146122	NA	NA	3.67097459	NA
Olenekian	NA	0.11826611	NA	NA	2.57805223	NA
Anisian	0.0737323	0.12273283	0.09999322	2.11020391	3.17294226	2.72344546
Ladinian	0.10877199	0.13086032	0.12029713	2.30554529	3.29815755	3.75491724
L. Carnian	NA	NA	0.12204885	NA	NA	3.54875186
U. Carnian	NA	NA	0.13903228	NA	NA	3.21130891
L. Norian	NA	NA	0.14027829	NA	NA	3.14123974
M. Norian	NA	NA	0.16783183	NA	NA	4.07636575
U. Norian	NA	NA	0.18671158	NA	NA	4.33655353
Rhaetian	NA	NA	0.17741984	NA	NA	4.38859378
Hettangian	NA	NA	0.18974149	NA	NA	4.66900132
Sinemurian	NA	NA	0.18521242	NA	NA	4.76454108
Pliensbachian	NA	NA	0.17210496	NA	NA	4.88224895
Toarcian	NA	NA	NA	NA	NA	NA

Supplementary Table 14. One-way PERMANOVA results for statistical significance of shape and functional morphospace changes through stage and epoch transitions. Abbreviation: Bonf., Bonferroni Corrected.

PERMANOVA of Stage Transitions						
Transitions	Shape			Function		
	P	$P^{Bonf.}$	F	P	$P^{Bonf.}$	F
Induan > Olenekian	0.511	1	0.8631	0.5591	1	0.7772
Olenekian > Anisian	0.0476	1	1.997	0.0498	1	2.292
Anisian > Ladinian	0.6695	1	0.7421	0.8269	1	0.4646
Ladinian > Lower Carnian	0.6698	1	0.7422	0.8782	1	0.3761
Lower Carnian > Upper Carnian	0.0123	1	2.823	0.0656	1	2.212
Upper Carnian > Lower Norian	0.9572	1	0.3515	0.9501	1	0.2209
Lower Norian > Middle Norian	0.7476	1	0.6261	0.3125	1	1.191
Middle Norian > Upper Norian	0.9945	1	0.2587	0.9989	1	0.05569
Upper Norian > Rhaetian	0.8055	1	0.4335	0.5248	1	0.8009
Rhaetian > Hettangian	0.5804	1	0.7746	0.7916	1	0.4803
Hettangian > Sinemurian	0.9573	1	0.3318	0.7218	1	0.4476
Sinemurian > Pliensbachian	0.0332	1	2.773	0.0519	1	2.976
Pliensbachian > Toarcian	0.163	1	1.561	0.0951	1	2.288
Total MANOVA	0.0001		1.931	0.0005		1.913
Total sum of squares:	2.879			1654		
Within-group sum of squares:	2.541			1461		
PERMANOVA Epoch Transitions						
Early Triassic > Middle Triassic	0.0223	0.1338	2.292	0.0222	0.1332	2.719
Middle Triassic > Late Triassic	0.0532	0.3192	1.933	0.1265	0.759	1.698
Late Triassic > Early Jurassic	0.1977	1	1.325	0.2324	1	1.34
Total MANOVA	0.0041		2.182	0.0268		1.958
Total sum of squares:	2.129			1216		
Within-group sum of squares:	2.038			1169		

Landmarking Regime:

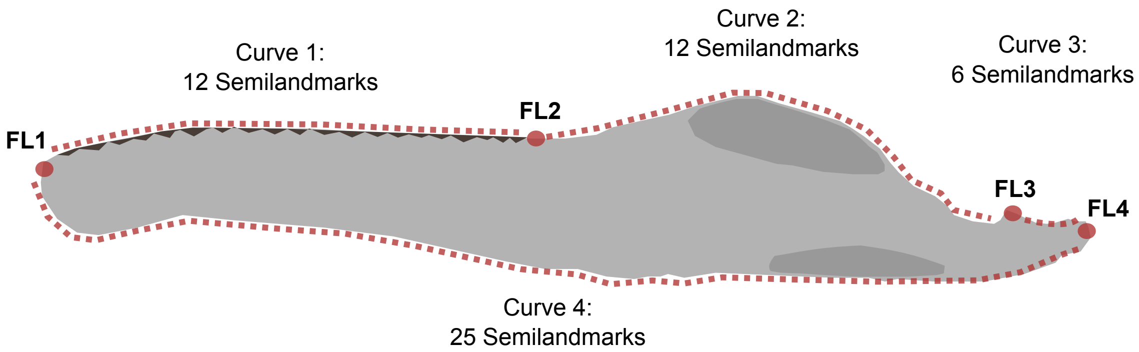

Features captured by semilandmark curves:

Curve 1:
Approximate measure of the tooth row and 'active functional surface' of the mandible.

Curve 2:
Approximate measure of the coronoid process and areas of adductor muscle attachment and the craniomandibular joint surface.

Curve 3:
Approximate measure of the retroarticular process.

Curve 4:
Approximate measure of total jaw curvature and areas of adductor muscle attachment.

Supplementary Figure 1. Geometric morphometric landmarking regime.. a). Landmarking Regime: FL = Fixed Landmarks: 1). Anterior-most tip of the mandible, 2). Posterior-most tip of the toothrow, 3). Beginning of the jaw articulation, 4). Posterior-most tip of the mandible.

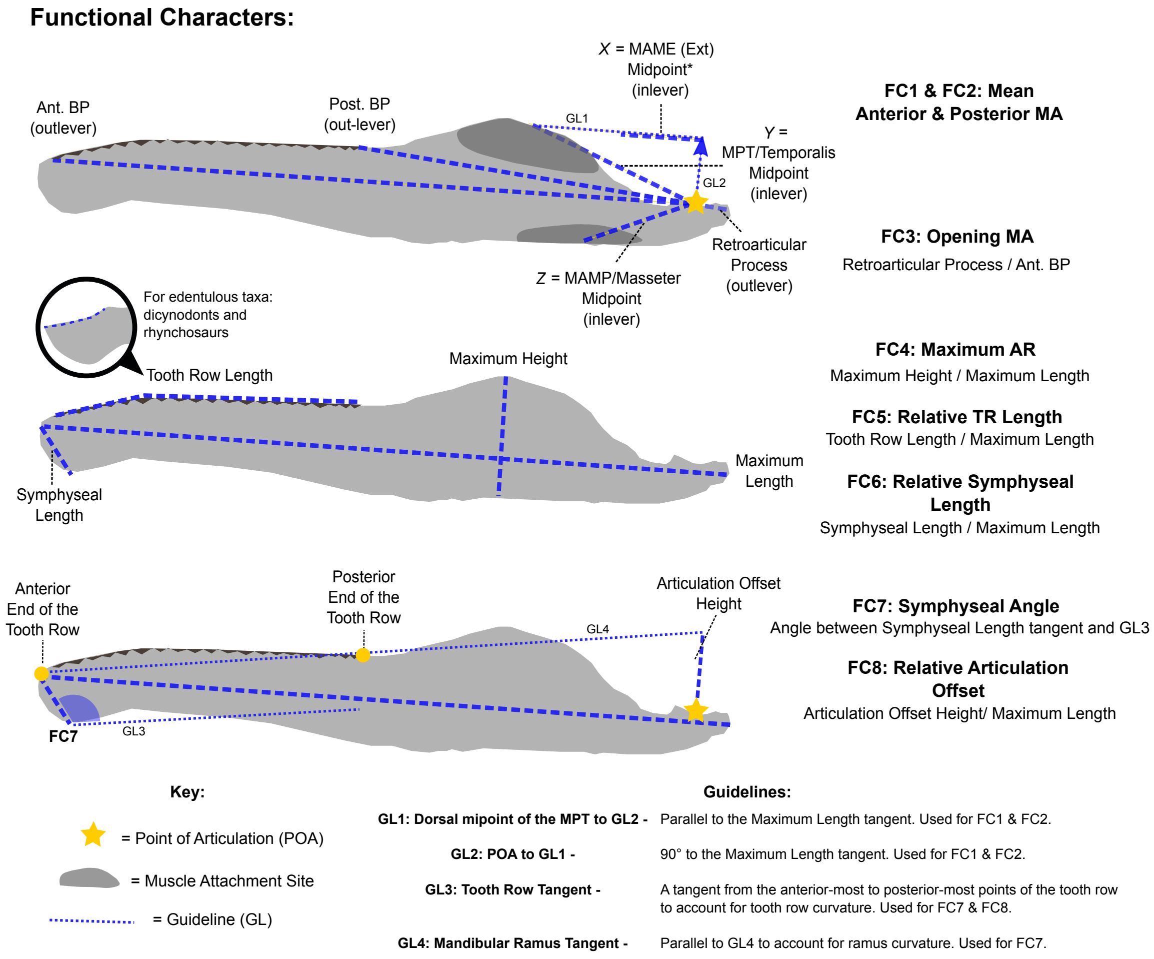

FC1 & FC2 (Mean anterior and posterior MA) Calculation = ((*X/BP* (Ant. Or Post.)* + *Y/BP* (Ant. Or Post.) + *Z/BP* (Ant. Or Post.))/3

Supplementary Figure 2. Functional character measurements. Functional Characters = FC: 1). Mean Anterior Mechanical Advantage, 2). Mean Posterior Mechanical Advantage, 3). Opening Mechanical Advantage, 4). Relative Maximum Aspect Ratio, 5). Relative Toothrow Length, 6). Relative Symphysis Length, 7). Symphyseal angle, 8). Relative Articulation Offset. *X not included for taxa with derived mammalian jaw joint, and mean calculation is adjusted accordingly. Abbreviations: Ant, Anterior. GL, Guideline. MA, Mechanical advantage. POA, Point of articulation. Post, Posterior.

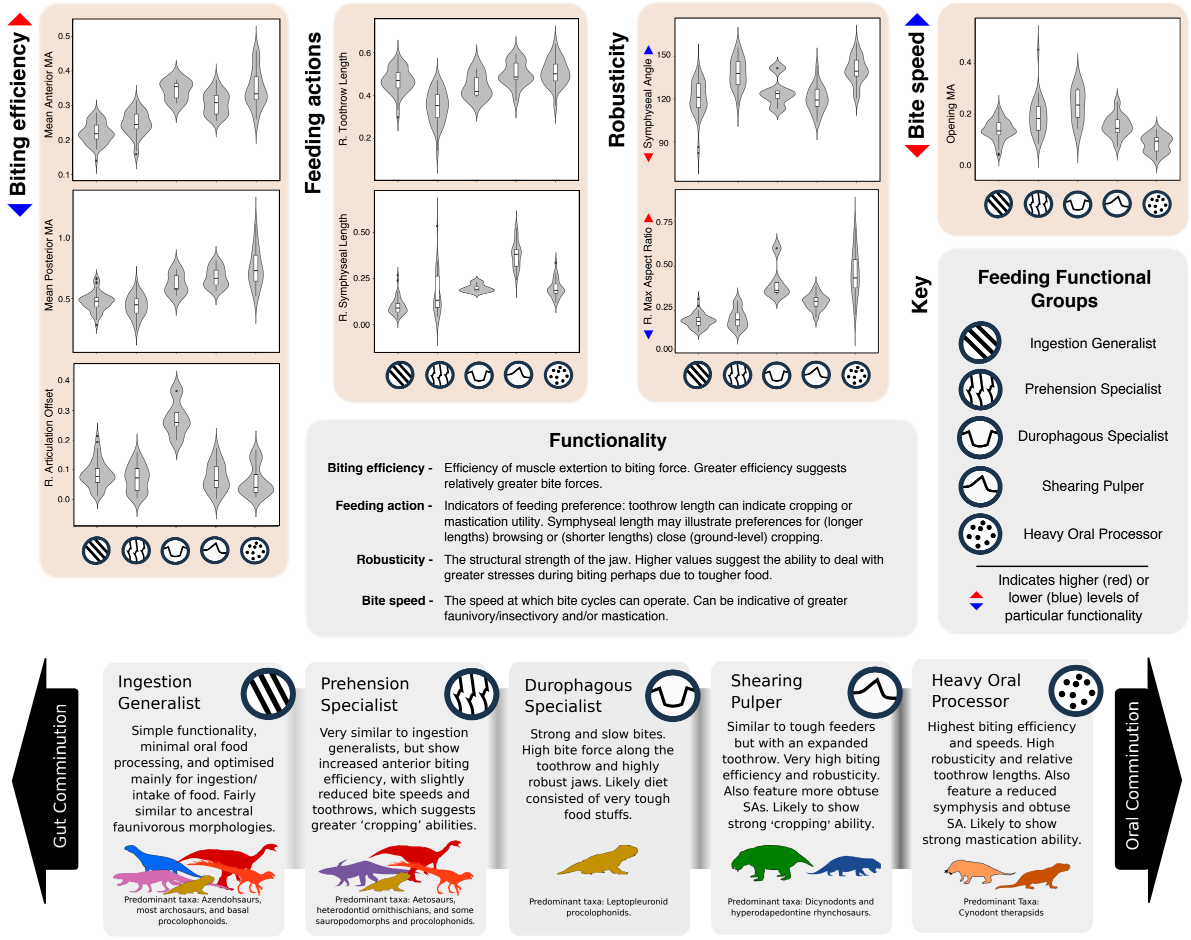

Supplementary Figure 3. Feeding Functional Group Characters – first step results. Feeding action characteristics not suitable for red/blue description as indicative of varying bite mechanics suggestive of different feeding. The strength of separation between the groups is illustrated by the darkness of the band connecting each FFG description box. Box plots showing median value (centre) and upper and lower quartiles representing the minimum and maximum bounds of the boxes, with whisker illustrating standard deviation. Abbreviations: MA, Mechanical advantage. R, Relative. N=136 taxa. All silhouettes created by S.S., but some are vectorised from artwork by Felipe Alves Elias (https://www.paleozoobr.com/) and Jeff Martz (United States National Park Service), available for academic use with attribution.

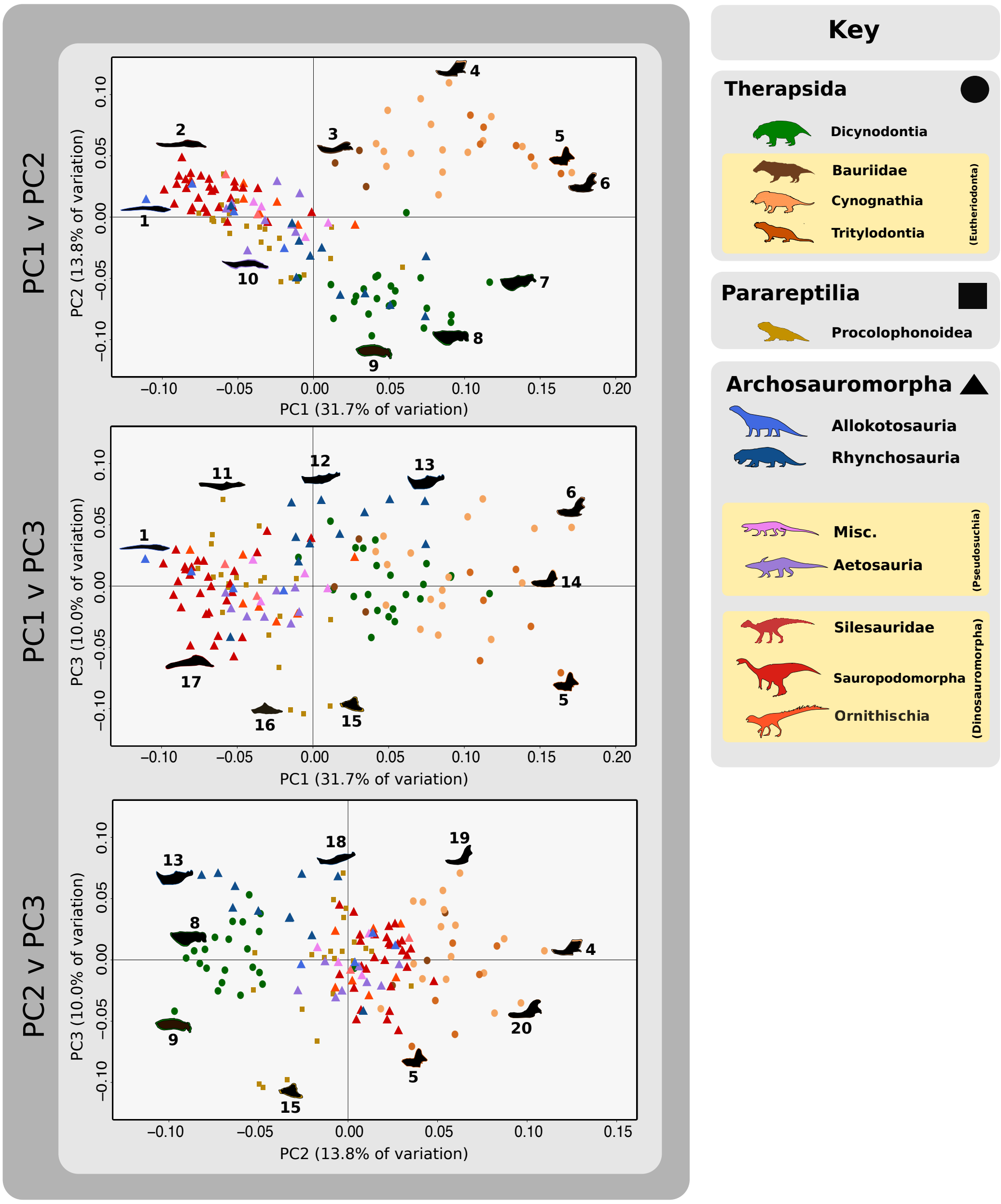

Supplementary Figure 4. Shape morphospaces using combinations of the first three principal components. Mandible silhouettes = 1. *Teraterpeton hrynewichorum*. 2. *Leyesaurus marayensis,* 3. *Traversodontoides wanguensis*. 4. *Sinognathus gracilis.* 5. *Yunnanodon brevirostre.* 6. *Siriusgnathus niemeyerorum*. 7. *Lystrosaurus hedini*, 8. *Ischigualastia jenseni*, 9. *Placerias hesternus*. 10. *Desmatosuchus haplocerus*, 11. *Kitchingnathus untabeni*, 12. *Fondonyx spenceri*, 13. *Hyperodapedon huxleyi*, 14. *Ruberodon roychowdhurii*, 15. *Hypsognathus_fenneri*. 16. *Leptopleuron lacertinum*, 17. *Sarahsaurus aurifontanalis*, 18. *Stenaulorhynchus sp*., 19. *Pascualgnathus polanskii*, 20. *Dadadon isaloi*. N=136 taxa. All silhouettes created by S.S., but some are vectorised from artwork by Felipe Alves Elias (https://www.paleozoobr.com/) and Jeff Martz (United States National Park Service), available for academic use with attribution.

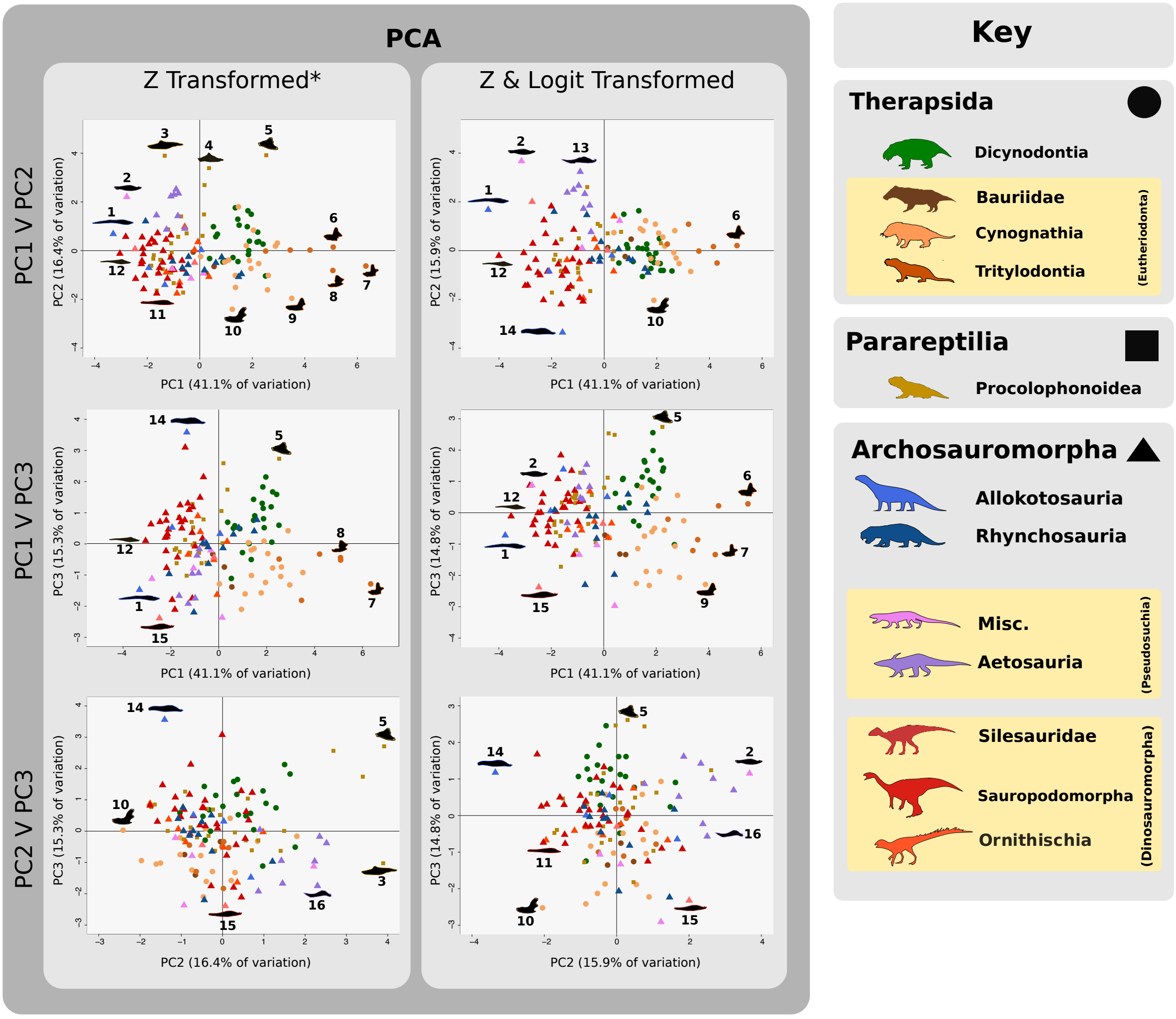

Supplementary Figure 5. Functional morphospace results from PCA using only z and z and logit transformed data. Mandible silhouettes = 1. *Teraterpeton hrynewichorum*. 2. *Lotosaurus adentus,* 3. *Eumetabolodon bathycephalus*. 4. *Leptopleuron lacertinum.* 5. *Hypsognathus_fenneri*. 6. *Yunnanodon brevirostre.* 7. *Bocatherium mexicanum*. 8. *Bienotherium yunnanense*. 9. *Ruberodon roychowdhurii*. 10. *Siriusgnathus niemeyerorum*. 11. *Eoraptor lunensis*. 12. *Pampadromaeus barberenai*. 13. *Stenomyti huangae.* 14. *Azendohsaurus madagaskarensis.* 15. *Silesurus opolensis*. 16. *Stagonolepis robertsoni.* N=136 taxa. All silhouettes created by S.S., but some are vectorised from artwork by Felipe Alves Elias (https://www.paleozoobr.com/) and Jeff Martz (United States National Park Service), available for academic use with attribution.

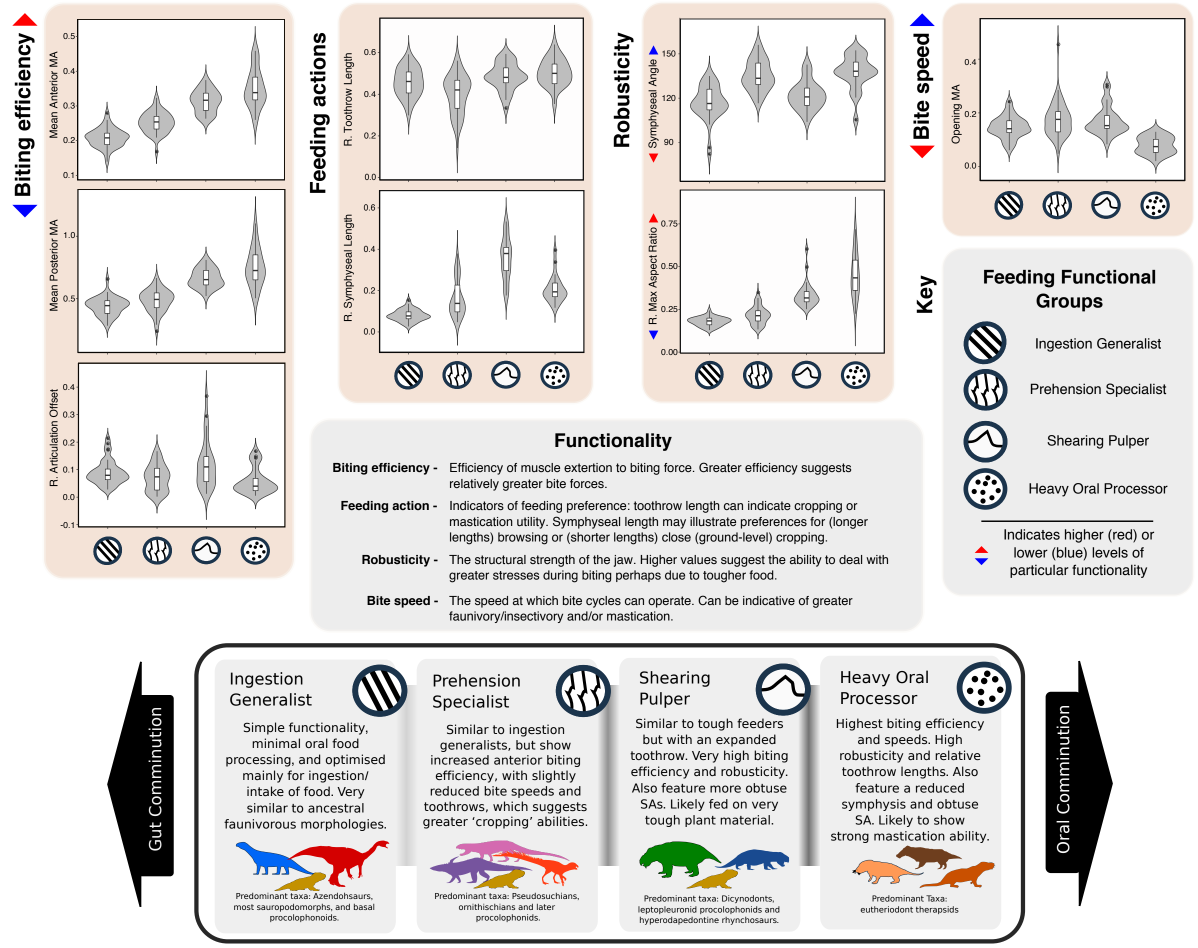

Supplementary Figure 6. Feeding Functional Group Characters for Logit data. Feeding action characteristics not suitable for red/blue description as indicative of varying bite mechanics suggestive of different feeding. The strength of separation between the groups is illustrated by the darkness of the band connecting each FFG description box. Box plots showing median value (centre) and upper and lower quartiles representing the minimum and maximum bounds of the boxes, with whisker illustrating standard deviation. Abbreviations: MA, Mechanical advantage. R, Relative. N=136 taxa. All silhouettes created by S.S., but some are vectorised from artwork by Felipe Alves Elias (https://www.paleozoobr.com/) and Jeff Martz (United States National Park Service), available for academic use with attribution.

Supplementary Note 2: R Code

1. Principal Component Analyses:

```
#Shape data (landmarks)
library(geomorph)
THbTr_GML.as<-arrayspecs(THbTr_GML.ds, 59, 2) #create 3d array from 2d landmark dataframe

plotAllSpecimens(THbTr_GML.as, mean = TRUE, links = NULL,) #pointscale = 0.5, meansize = 1) ### plot all specimens
PCA<-plotTangentSpace(THbTr_GML.as, warpgrids=T,) #label=TRUE, verbose=TRUE) ### make a simple morphospace of pc1 and pc2

PCA$pc.summary  ### variation on each axis
PCA$pc.scores   ### pc scores for each taxon on each morphospace axis
GMPCscores<-PCA$pc.scores #create PC scores object

#Functional data
library(FactoMineR)
THbTr_Fm<-read.csv(#read in raw functional data)
Thbt.pca.Zsc<-PCA(THbTr_Fm, scale.unit=T, ncp=10) #PCA of raw data includes z transformation (no logit transformation)
```

2. Cluster analyses:

```
library(factoextra)
Fdata<- #Read in the functional measurement data
Fdata<-scale(Ing_Gen_dat, center=T, scale=T) #scale and center the data

#Hierarchical Analysis - Exploratory.
FMres.hc <- eclust(Fdata, FUNcluster = "hclust",  k.max=10, hc_metric="euclidean", hc_method="ward.D2", nboot=4000)
#Specify range of cluster numbers to consider classifying taxa into.
#Cluster range set as used in analysis for ingestion generalists).
FMres.hc.IG_RV <- eclust(Fdata, FUNcluster = "hclust", k.max=2,3,4,5,6,7,8, hc_metric="euclidean", hc_method="ward.D2", nboot=4000)

FMres.hc
FMres.hc_$cluster #View cluster assignments for each taxon
FMres.hc.PG_RV$cluster

plot(FMres.hc, cex = 0.5)
plot(FMres.hc.IG_RV, cex = 0.5)

###########Partition Cluster Methods
#K-means Analysis #for PAM, in FUNcluster replace “kmeans” with “pam”
##Cluster range set as used in analysis for all taxa.
FMres.Km<-eclust(Fdata, FUNcluster = "kmeans", k.max=4,5,6,7,8, nstart=200, nboot=4000)
#Cluster range set as used in analysis for ingestion generalists.
FMres.Km.PG_RV<-eclust(Fdata, FUNcluster = "kmeans", k.max=2,3,4,5,6,7,8, nstart=200, nboot=4000)

FMres.Km
FMres.Km$cluster #View cluster assignments for each taxon
```

3. Disparity analysis:

```
#######Additional Functions#

## This function isolates one half of a distance matrix
upperTriangle<-function (x, diag = FALSE, byrow = FALSE)
{
  if (byrow)
    t(x)[rev(upper.tri(x, diag = diag))]
  else x[upper.tri(x, diag = diag)]
}


## This function calculates the mean pairwise distance within a bin and
runs bootstrapping to create error bars around mean
bootstrapMPD <- function(dissim) {

  dissim <- upperTriangle(dissim)

  mean <- mean(dissim, na.rm=TRUE)

  Z <- length(dissim[complete.cases(dissim)])

  boot.mean <- vector()

  for(i in 1:10000) {
    boot.mean[i] <-
mean(dissim[complete.cases(dissim)][sample.int(Z,Z,replace=TRUE)])
  }

  #Lower 0.05 for the mean
  lower <- sort(boot.mean)[length(boot.mean)*0.05]

  #Upper 0.95 for the mean
  upper <- sort(boot.mean)[length(boot.mean)*0.95]

  return(cbind(mean,lower,upper))

}

### This function can extract certain groups from distance matrix
extractPairwise <- function(dMat, listGroups) {

  dMatNames <- rownames(dMat)
  outputList <- c()
  for(i in 1:length(listGroups)) {
    rowColName <- match(listGroups[[i]], dMatNames)
    rowColName <- rowColName[complete.cases(rowColName)]
    outputList[[i]] <- dMat[rowColName, rowColName]
  }
  names(outputList) <- names(listGroups)
  return(outputList)
}

###Load data - distance matrices of shape or standardised functional data
#######Assign data
proc.dist <- #read.csv(Distance matrix object)
head(proc.dist)
dim(proc.dist)

#assign taxa to bin based on taxon ranges
#Taxa MUST be in same order and names must match exactly!
taxon.ranges <- read.table("taxon.ranges.txt", header=T, row.names=1)
```

```
# Rearrange taxon ages object to match the order of the procustes distances
object
# The proc dist object is usually ordered based on the sequence which the
landmarks were acquired
taxon.ranges <- as.data.frame(taxon.ranges[rownames(proc.dist),])
dim(taxon.ranges)
taxon.ranges # does this still look OK?

# are the row names of the taxon ages and dissim matrix identical?
identical(rownames(taxon.ranges), rownames(proc.dist))

# Need object denoting the range of time bins to place taxa in
bin.ranges <- #read.table(timebin ranges)
bin.ranges

# Assign the taxa to bins based on the range and dates, in the object
'taxon.bins'
taxon.bins <- list()
for (i in 1:length(rownames(bin.ranges))) {taxon.bins[[i]] <-
rownames(taxon.ranges)[which(taxon.ranges$FAD > bin.ranges[i,"min.age"] &
taxon.ranges$LAD < bin.ranges[i,"max.age"])]}
names(taxon.bins) <- rownames(bin.ranges)
taxon.bins # view the object, does it look OK?

# note the taxon bins object could be time bins or clade bins etc, to make
clade file you would need to use different code to make list
# something like
# inputList <- list(group_1_list, group_2_list,group_3_list ,group_4_list)
# names(inputList) <- c("group_1", "group_2", "group_3", "group_4")
# inputList # does this look correct, are the correct names in the correct
group?


### Within bin Mean Pairwise Procruestes Distances
# Using the function we made earlier 'extractPairwise' we will now create a
separate Procruestes distances object object for each time bin
binned.proc.dist <- extractPairwise(proc.dist, taxon.bins)
binned.proc.dist # this is a large object with a distance matrix for each
time bin

binned.proc.dist[[1]] # view the distance object for time bin 1
# you could always save the original full distance matrix outside of R and
check to see that the distances in there are the same as those in these bin
subsets

# Now we will use the bootstrapMPD function to calculate the mean
Procrustes distance between taxa in each time bin that we have

# Make empty results object to save
MPD.results <-  matrix(NA, nrow=length(taxon.bins), ncol=3)

# for each time bin, it will calculated the MPD for the taxa within it
for(i in 1:length(taxon.bins)) {
  MPD.results [i,] <- bootstrapMPD(binned.proc.dist[[i]])
}

colnames(MPD.results) <- c("mean", "lower", "upper")
rownames(MPD.results) <- names(taxon.bins)
MPD.results
```

Supplementary References:

1. Button, D. J., & Zanno, L. E. Repeated evolution of divergent modes of herbivory in non-avian dinosaurs. *Curr. Biol.* **30**, 158-168 (2020).
2. Button, D. J., Rayfield, E. J., & Barrett, P. M. Cranial biomechanics underpins high sauropod diversity in resource-poor environments. *Proc. R. Soc. B Biol. Sci.* **281**, 20142114 (2014).
3. Button, D. J., Barrett, P. M., & Rayfield, E. J. Craniodental functional evolution in sauropodomorph dinosaurs. *Paleobiology* **43**, 435-462 (2017).
4. Anderson, P. S., Friedman, M., Brazeau, M. D., & Rayfield, E. J. Initial radiation of jaws demonstrated stability despite faunal and environmental change. *Nature* **476**, 206–209 (2011).
5. Stubbs, T.L., Pierce, S.E., Rayfield, E.J. & Anderson, P.S. Morphological and biomechanical disparity of crocodile-line archosaurs following the end-Triassic extinction. *Proc. R. Soc. B Biol. Sci.* **280**, 20131940 (2013),
6. MacLaren, J. A., Anderson, P. S., Barrett, P. M., & Rayfield, E. J. Herbivorous dinosaur jaw disparity and its relationship to extrinsic evolutionary drivers. *Paleobiology* **43**, 15-33 (2017).
7. Warnes, G. R., Bolker, B., & Lumley, T. gtools: Various R Programming Tools. R package version 3.8.2. (2020).
8. Benevento, G. L., Benson, R. B. J., & Friedman, M. Patterns of mammalian jaw ecomorphological disparity during the Mesozoic/Cenozoic transition. *Proc. R. Soc. B Biol. Sci.* **286**, 1902 (2019).
9. Warton, D. I., & Francis K. C. H. The arcsine is asinine: the analysis of proportions in ecology. *Ecology* **92**, 3-10 (2011).
10. Beck C., Lu B., & Greevy R. nbpMatching: Functions for Optimal Non-Bipartite Matching. R package version 1.5.1. (2016).
11. Oksanen, J., F. G. Blanchet, M. Friendly, R. Kindt, P. Legendre, D. McGlinn, P. R. Minchin et al. "vegan: Community Ecology Package. R package version 2.5. 4. 2019." (2019).
12. Westneat, M. W. Transmission of force and velocity in the feeding mechanisms of labrid fishes (Teleostei, Perciformes). *Zoomorphology*, **114,** 103-118 (1994).
13. Westneat, M. W. Evolution of levers and linkages in the feeding mechanisms of fishes. *Integr. Comp. Biol.*, **44,** 378-389 (2004).
14. Stayton, C. T. Testing hypotheses of convergence with multivariate data: morphological and functional convergence among herbivorous lizards. *Evolution*, **60**, 824-841 (2006).
15. Wainwright, P. C., & Richard, B. A. Predicting patterns of prey use from morphology of fishes. *Environ. Biol. Fishes*, **44**, 97-113 (1995).
16. Anderson, P. S., & Westneat, M. W. Feeding mechanics and bite force modelling of the skull of Dunkleosteus terrelli, an ancient apex predator. *Biol. Lett.,* **3**, 77-80 (2006).

17. Reisz, R. R. & Sues, H. D. Herbivory in late Paleozoic and Triassic terrestrial vertebrates. *Evolution of Herbivory in Terrestrial Vertebrates: Perspectives from the fossil record* (eds Sues, H. D.), 9-41. (Cambridge Univ. Press, Cambridge, 2000)
18. Daegling, D. J. Biomechanical scaling of the hominoid mandibular symphysis. *J. Morphol.*, **25**, 12-23 (2001).
19. Jones, M. E., O'higgins, P., Fagan, M. J., Evans, S. E., & Curtis, N. Shearing mechanics and the influence of a flexible symphysis during oral food processing in Sphenodon (Lepidosauria: Rhynchocephalia). *Anat. Rec.,* **295**, 1075-1091 (2012).
20. Porro, L. B., Holliday, C. M., Anapol, F., Ontiveros, L. C., Ontiveros, L. T., & Ross, C. F. Free body analysis, beam mechanics, and finite element modeling of the mandible of *Alligator mississippiensis*. *J. Morphol.*, **272**, 910-937 (2011).
21. Walmsley, C.W., et al. Why the long face? The mechanics of mandibular symphysis proportions in crocodiles. *PLoS One*, **8**, e53873 (2013).
22. Janis, C. Correlations between craniodental morphology and feeding behavior in ungulates: reciprocal illumination between living and fossil taxa. *Functional Morphology in Vertebrate Paleontology* (Eds Thomason J. J.), 76-98 (Cambridge Univ. Press, Cambridge, 1995).
23. Ramsay, J. B., & Wilga, C. D. Morphology and mechanics of the teeth and jaws of white-spotted bamboo sharks (*Chiloscyllium plagiosum*). *J. Morphol.* **268**, 664-682 (2007).
24. Benton, M. J., Ruta, M., Dunhill, A. M., & Sakamoto, M. The first half of tetrapod evolution, sampling proxies, and fossil record quality. *Palaeogeogr. Palaeoclimatol. Palaeoecol.* **372**, 18-41, (2013).
