# Supplementary material for: Niche partitioning shaped herbivore macroevolution through the early Mesozoic
Source: Nat Commun. 2021 May 14;12:2796. doi: 10.1038/s41467-021-23169-x (PMC8121902; doi:10.1038/s41467-021-23169-x)
Supplement: Supplementary file 2 — Description of Additional Supplementary Files [file 41467_2021_23169_MOESM2_ESM.pdf]

Supplementary Data Legends

Supplementary Data 1. Procrustes-aligned Landmark data. The geometric morphometric Procrustes-aligned landmark data used to assess mandibular shape variation.

Supplementary Data 2. Raw functional measurement data. The raw functional character data used to assess mandibular functional variation.

Supplementary Data 3. Shape PC Scores. The principal component (PC) scores derived from a principal component analysis of the Procrustes aligned landmark data. (Source data used in Figs. 1,5 and 6).

Supplementary Data 4. Functional PC Scores. The principal component (PC) scores derived from a principal component analysis of the z transformed functional data. (Source data used in Figs. 1,5 and 6).

Supplementary Data 5. Cluster analysis results and functional feeding group (FFG) assignments. The groups identified by the three (hierarchical, K-means, and partition around the medioid) sets of cluster analysis of the z transformed data. (Source data for Figs. 2 and 3).

Supplementary Data 6. Ingestion generalist cluster analysis results and sub-FFG assignments. The groups identified by the three (hierarchical, K-means, and partition around the medioid) sets of cluster analysis of the ingestion generalist subset of the z transformed data to identify sub-functional feeding groups (FFG). (Source data for Figs. 2 and 3).

Supplementary Data 7. Feeding functional group functional characteristics. Functional characteristics for each taxon, arranged by each feeding functional group generated using the z transformed data. (Source data for Fig. 2).

Supplementary Data 8. Taxa timebin and FFG designations. Taxa included in each timebin with subclade and feeding functional group (FFG) classification. (Source data for Fig. 3).

Supplementary Data 9. Feeding functional groups through time. Overall clade and feeding functional group (FFG) abundance through time summary. (Source data for Fig. 3).

Supplementary Data 10. Early Mesozoic assemblage data. Overall clade and feeding functional group (FFG) abundance across different assemblages through the early Mesozoic. (Source data for Fig. 4).

Supplementary Data 11. Feeding functional groups per assemblage. Herbivore cooccurrences by assemblage with clade and feeding functional group (FFG) classifications to identify potential competitive conflict. (Source data for Fig. 4).

Supplementary Data 12. Reference list for all taxon images. References for the (literature) sources for all images used in this study.

Supplementary Data 13. First and last appearance dates for all taxa. First and last appearance dates for all taxa used in this study.

Supplementary Data 14. External cluster validation groups. Clade groupings used during the external cluster validation tests to identify the degree of correlation between overall clade and cluster groupings.

Supplementary Data 15. Logit cluster groups. The groups identified by the three (hierarchical, K-means, and partition around the medioid) sets of cluster analysis of the logit transformed data.

Supplementary Data 16. Logit feeding functional group functional characteristics.
Functional characteristics for each taxon, arranged by each feeding functional group generated using the logit transformed data. (Source data for Fig. S6).
